# Collaborating genomic, transcriptomic and microbiomic alterations lead to canine extreme intestinal polyposis

**DOI:** 10.18632/oncotarget.25646

**Published:** 2018-06-26

**Authors:** Jin Wang, Tianfang Wang, Micah A. Bishop, John F. Edwards, Hang Yin, Stephen Dalton, Laura K. Bryan, Shaying Zhao

**Affiliations:** ^1^ Department of Biochemistry and Molecular Biology, Institute of Bioinformatics, University of Georgia, Athens, GA 30602, USA; ^2^ Department of Veterinary Pathobiology, College of Veterinary Medicine & Biomedical Sciences, Texas A&M University, College Station, TX 77843, USA; ^3^ Department of Small Animal Clinical Sciences, College of Veterinary Medicine & Biomedical Sciences, Texas A&M University, College Station, TX 77843, USA

**Keywords:** canine extreme intestinal polyposis, germline and somatic mutation, MYC network activation, microbiome, B. uniformis and redox

## Abstract

Extreme intestinal polyposis in pet dogs has not yet been reported in literature. We identified a dog patient who developed numerous intestinal polyps, with the severity resembling human classic familial adenomatous polyposis (FAP), except the jejunum-ileum junction being the most polyp-dense. We investigated this dog, in comparison with 22 other dogs with spontaneous intestinal tumors but no severe polyposis, and with numerous published human cancers. We found, not *APC* mutation, but three other alteration pathways as likely reasons of this canine extreme polyposis. First, somatic truncation mutation W411X of FBXW7, a component of an E3 ubiquitin ligase, over-activates MYC and cell cycle-promoting network, accelerating crypt cell proliferation. Second, genes of protein trafficking and localization are downregulated, likely associated with germline mutation G406D of STAMBPL1, a K63-deubiquitinase, and MYC network activation. This inhibits epithelial apical-basolateral polarity establishment, preventing crypt cell differentiation. Third, *Bacteroides uniformis*, a commensal gut anaerobe, thrives and expresses abundantly thioredoxin and nitroreductase. These bacterial products could reduce oxidative stress linked to host germline mutation R51X of CYB5RL, a cytochrome b5 reductase homologue, decreasing cell death. Our work emphasizes the close collaboration of alterations across the genome, transcriptome and microbiome in promoting tumorigenesis.

## INTRODUCTION

Canine cancers represent one of the best animal models of human cancers [1–4], because of the shared biology (e.g., intact immune system), physiology, living environment and clinical symptoms between the two species. Indeed, genomic studies from our group and others have revealed a high degree of molecular homology for histopathologically matched cancer types/subtypes between the dog and the human [5–7]. For example, the stepwise model of human colorectal tumorigenesis [8] also applies to spontaneous colorectal tumors in pet dogs [5, 9]. Furthermore, our group has successfully developed a novel dog-human comparative genomics and oncology strategy for driver-passenger discrimination, a central aim of cancer research [10], for colorectal cancer (CRC) copy number alteration [11, 12].

In humans, individuals with classic familial adenomatous polyposis (FAP) differ from the general population [13, 14]. These individuals develop significantly more (>100) adenomatous polyps in their colon, beginning at much younger age (age 16 on average). They also develop polyps in the small intestine and other places [15, 16]. The underlying pathogenic mechanism is well studied [8, 13, 14]. Most classic FAP patients inherit a dysfunctional copy of the *APC* gene, and then, a second mutation inactivates the other functional copy of *APC*. This results in translocation of β-catenin into the nucleus, activating WNT/β-catenin targets (e.g., *MYC*) [17] and accelerating cell proliferation. Furthermore, defective *APC* also interferes with cell adhesion, cytoskeleton and the establishment of epithelial apical-basolateral polarity. All these lead to extreme colon polyposis. Other variants of FAP include attenuated FAP, which is also *APC* mutation-associated but the patients typically develop polyps at older age, and autosomal recessive FAP, which is *MUTYH* mutation-associated and the patients develop fewer polyps. Hereditary nonpolyposis colorectal cancer (HNPCC), another inherited condition, is caused by mutations of DNA mismatch repair genes [13, 14, 18–20] and others [21].

Extreme intestinal polyposis in pet dogs has not yet been reported in literature, and the underlying pathogenic mechanism is unknown. We are fortunate to identify such a case. We set out to molecularly characterize this rare canine condition and compare our findings with those of human studies, as described below.

## RESULTS

### N14-77 represents a rare canine case of extreme intestinal polyposis

**Figure 1:**
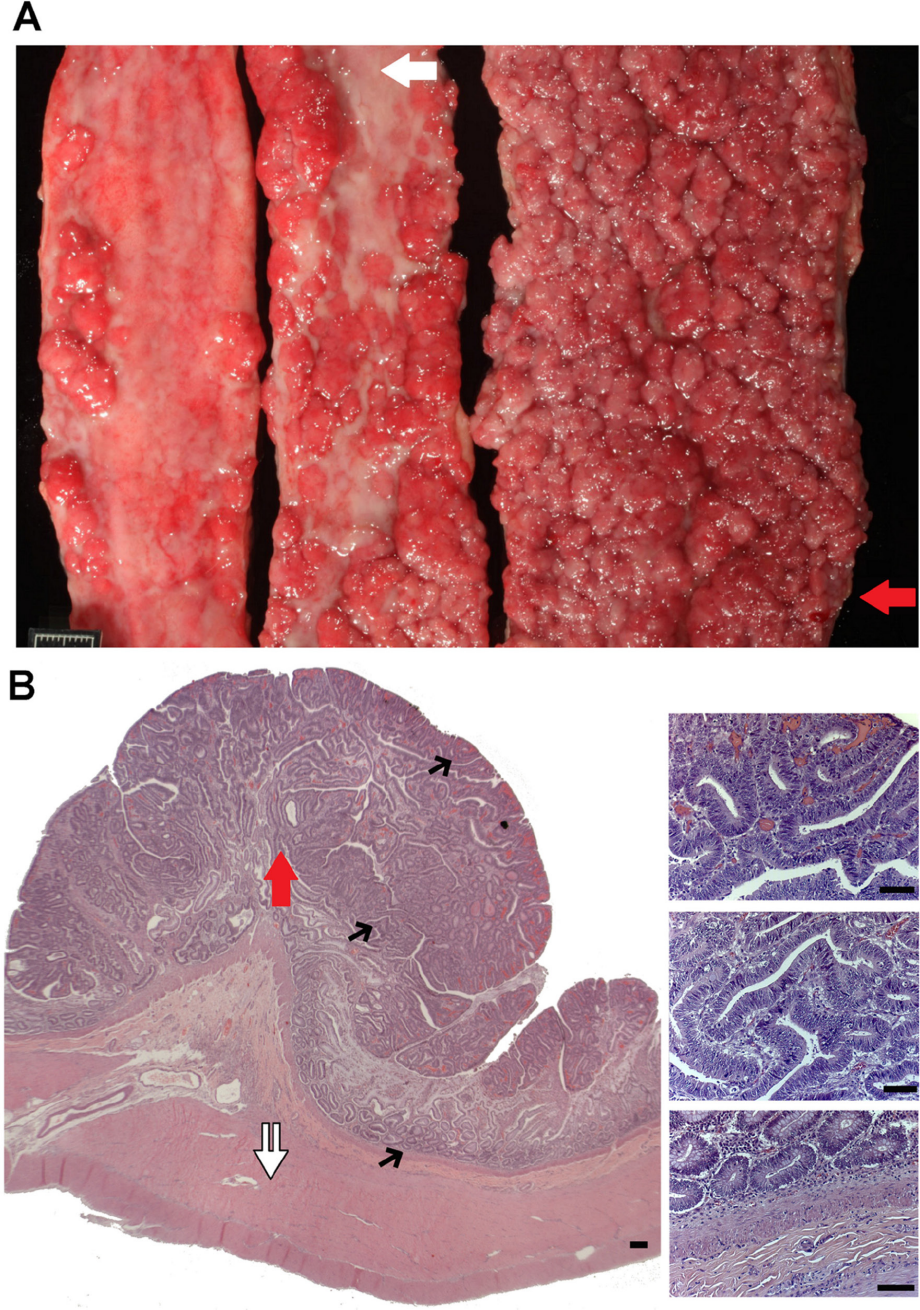
N14-77 represents a rare case of extreme intestinal polyposis in the dog **(A)** Opened small intestinal segments from left to right are from the proximal jejunum, middle jejunum and distal jejunum-ileum junction, respectively. The red arrow indicates the area used for polyp dissection and sequencing (WGS and RNA-seq). The white arrow illustrates an unaffected inter-polyp region used for normal sample WGS. The scale bar is 1cm-long. **(B)** Representative H&E images of the distal jejunum-ileum junction indicate extensive cell proliferation and no invasion of proliferating enterocytes into the lamina propria or submucosa. The white double arrow exemplifies unaffected submucosa and muscularis propria tissue being dissected for normal sample RNA-seq. Images on the right are blowups of the corresponding sites pointed by black arrows on the left. Scale bar, 50μm.

A rare canine case of extreme intestinal polyposis (Figure 1) was diagnosed at the Texas A&M University Veterinary Medical Teaching Hospital, and assigned “N14-77” as the case identifier. The detailed case information is provided in Supplementary Information and summarized below.

At presentation, the N14-77 patient, a 9-year-old neutered male dog of Golden Retriever-mix, had a two-month history of blood-tinged, watery diarrhea and was in poor body condition. Complete blood count revealed a microcytic, hypochromic, regenerative anemia with a severe neutrophilia and hypoalbuminemia. Abdominal radiographs and ultrasounds indicated extensive intestinal changes. A rectal scraping found numerous, degenerate neutrophils containing phagocytosed bacteria and small yeast.

Euthanasia was selected. A full necropsy indicated that, while no significant abnormalities in other organ systems, about 70% of the small intestinal mucosa was affected. Specifically, intestine, extending primarily from the mid-jejunum to the ileocecal junction, was severely thickened by innumerable, 3 mm to 1.1 cm, firm nodules that progressively coalesced into large, plaque-like, 10-30 cm-long areas with a red, granular surface. The most severe region located at the distal jejunum-ileum junction (Figure 1A).

Histologic examination indicated numerous single to coalescing polyps within the mucosa of sections from the jejunum to the proximal colon, and the epithelium from crypts to mucosal surface was uniformly hyperplastic (Figure 1B). The mucosa comprising the inter-polyp regions and within the distal colon also displayed mild to moderate hyperplasia, with variable neutrophilic infiltration and mild enterocolitis. Notably, neither malignant neoplastic transformation of nor invasion of the lamina propria by enterocytes lining the intestinal villi, crypts, or colonic glands was observed (Figure 1B).

Except for the location (extending primary from the mid-jejunum to the ileocecal junction and with the distal jejunum-ileum junction being the most affected), the severity of polyposis in N14-77 resembles classic FAP patients in humans.

### We performed whole genome sequencing (WGS) and RNA-seq

To characterize N14-77, we performed WGS and RNA-seq analyses with frozen polyp and normal (or rather unaffected) samples. To maximally identify molecular changes associated with extreme intestinal polyposis, we chose polyps dissected from the most affected and polyp-dense area, located at the distal jejunum-ileum junction (Figure 1A), for polyp WGS and RNA-seq. Hence, the findings represent multiple polyps but not individual ones. As controls, we performed WGS with unaffected tissue dissected from one of the inter-polyp regions of the mid-jejunum (Figure 1A), as well as RNA-seq with unaffected submucosa and muscularis propria tissue dissected away from the polyp-dense mucosa used in polyp-sequencing (Figure 1B). Thus, WGS and RNA-seq normal samples differ in their locations.

For WGS, we generated a 15X sequence coverage for the polyp sample and a 13X sequence coverage for the normal sample, with a fragment coverage at approximately 21X (Supplementary Table 1A). For RNA-seq, we acquired about 80 million paired-reads for the polyp sample and 74 million paired-reads for the normal sample (Supplementary Table 1B). For comparison, we also performed WGS and/or RNA-seq analyses with 26 intestinal normal or tumor samples from 22 dogs with spontaneous intestinal tumors, along with a healthy dog (Supplementary Table 1C). Differing from N14-77, none of these dogs have this extreme polyposis phenotype. We developed a pipeline (Supplementary Figure 1) to interrogate the data.

### We corrected genomic sequence errors in the canine *APC* gene

**Figure 2:**
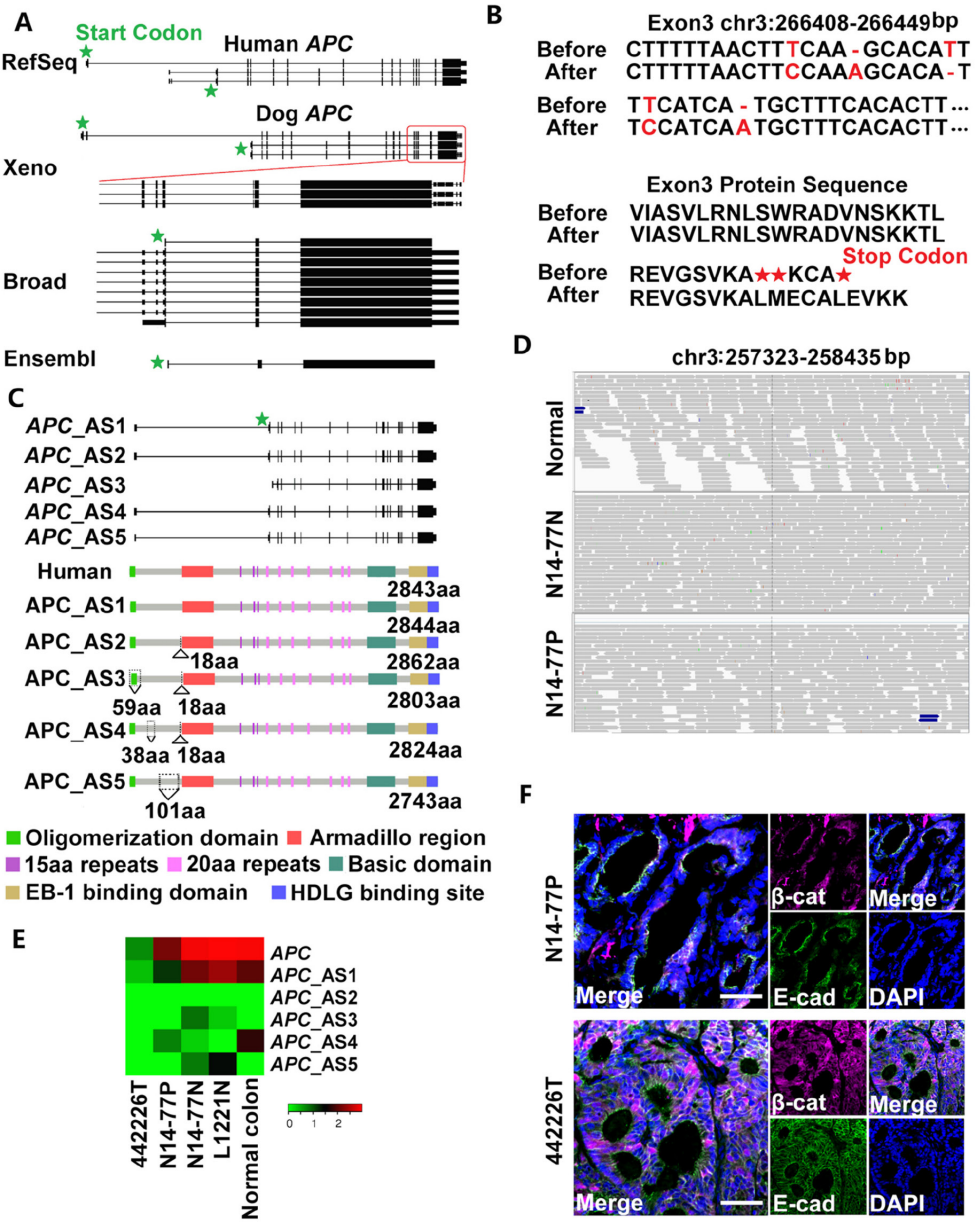
Neither germline nor somatic mutations of *APC* were found in N14-77 **(A)** Canine *APC* is annotated inconsistently. “Xeno” represents human xenoRefGene (mapping human transcript or protein sequences to the canine genome). For Broad annotation [22], only the 3'–end portion enclosed by the red square is shown. Each line designates a transcript, with coding exons, UTR exons and introns respectively represented by tall bars, short bars and the lines between the bars. **(B)** Five sequence errors (red) were uncovered in exon 3 of *APC* in the canine genome assembly canFam3.1 (top), resulting in premature stop codons (bottom). Before and After: before and after error correction. **(C)** Five alternatively spliced (AS) transcripts and protein isoforms were identified after the error correction in B. Also shown is the canonical isoform of human APC, with domains indicated. Amino acid (aa) insertions and deletions are indicated by ∆ and ∇, respectively. **(D)** Representative IGV images show no convincing mutations in *APC*. The canine genomic region shown corresponds to the human site (codons 1061-1431) that harbors some of *APC* mutation hotspots. **(E)** The heatmap indicates the *APC* expression level in log_2_ (*FPKM*) (fragments per kilobase of exon per million fragments mapped). “L1221N” and “Normal colon” are normal colon epithelial tissues from two dogs, while “442226T” is a colorectal tumor with *APC* deletion from another dog. (**F)** Representative confocal images indicate no nuclear enrichment of β-catenin (β-cat) in N14-77 polyps, unlike 442226T (with *APC* deletion). Scale bar, 50μm.

Because *APC* mutations characterize the human FAP condition, our initial hypothesis was that *APC* is mutated in N14-77. We hence first investigated canine *APC*, but noted that it is annotated inconsistently. In the Ensembl database, *APC* consists of only three exons, unlike its human counterpart which has 18 exons (Figure 2A). In the Broad annotation [22], *APC* has more exons but only three are coding (Figure 2A). This again differs from human *APC*, which has 14-16 coding exons among its transcripts (Figure 2A). The human “xenoRefGene” annotation shows better resemblance (Figure 2A). This however has been achieved by mapping human APC sequences onto the dog genome, not using dog-specific data.

To resolve this inconsistency, we examined our WGS and RNA-seq reads that are mapped onto the canine *APC* locus. These reads are from normal and tumor intestinal tissues of 23 dogs (Supplementary Table 1C), in addition to N14-77, and from other canine tissues [6, 7]. We detected 5 sequence errors in exon 3 of *APC* in the canine reference genome [1], including two base substitutions, two base deletions and one base insertion (Figure 2B; Supplementary Figure 2). These errors result in premature stop codons and mis-annotation of *APC* in Broad and Ensembl databases (Figure 2A).

After removing the errors, we remapped our RNA-seq reads (Supplementary Figure 2) and reassembled the canine *APC* gene and transcripts. Five transcripts were identified, with 16-17 exons in total of which 15-16 are coding (Figure 2C), better matching their human counterparts. The transcripts yield slightly different protein isoforms. One isoform is nearly identical to the canonical human APC protein (Supplementary Table 2B). The other isoforms have insertions or deletions, all occurring before the armadillo repeat domain (Figure 2C).

### We found neither germline nor somatic *APC* mutations

To discover *APC* mutations in N14-77, we investigated WGS and RNA-seq reads, individually or combined, of polyp and normal samples. The combined sequence coverage reaches to 136X on average for APC coding regions (Supplementary Table 2C). We used popular software tools, GATK for germline- and MuTect for somatic mutation discovery, with the corrected *APC* genomic sequence (Figure 2B). Surprisingly, neither germline nor somatic mutations were detected. To confirm this, we manually examined sequence read alignment of each of the 18 *APC* exons (Figure 2C) with IGV, a widely-used genomics viewer. No convincing mutations were noticed. None of the changes are significantly recurrent, with most found in one or two reads (Figure 2D; Supplementary Figure 3).

Given the prominence of *APC* mutation in human FAP, our result is somewhat unexpected. To determine if *APC* alters via other mechanisms, we examined its expression and found no significant alteration (Figure 2E). This is because the N14-77 normal sample expresses a comparable level of *APC*_AS1, which corresponds to the canonical human APC (Supplementary Table 2A), as normal colon samples of other dogs. N14-77 polyps express a lower amount of *APC*_AS1, but the level is still higher than colorectal tumors with *APC* deletion (Figure 2E). Notably, we did not detect significant nuclear enrichment of β-catenin in N14-77 polyp cells (Figure 2F), which does not support APC inactivation.

Besides *APC*, we also investigated other genes with germline mutations involved in human colorectal tumor development. These include *AXIN2*, *BMPR1A*, *GREM1*, *MUTYH*, *PTEN*, *SMAD4* and *STK11*, as well as others (e.g., DNA mismatch repair genes) [13, 14, 18–21]. We examined both coding regions, with average sequence coverages ranging 136-615X after combining WGS and RNA-seq reads of both polyp and normal samples (Supplementary Table 2C), as well as their promoters (22-45X sequence coverage; see Supplementary Table 2C). We only detected two germline mutations: L588P of AXIN2 and A161V of MUTYH. Both mutations have however occurred during evolution (Supplementary Table 2C) and thus are most likely natural variants - not pathogenic. We also detected three germline mutations in the *AXIN2* promoter and two germline mutations in the *MUTYH* promoter. We however did not identify any transcription factor binding sites that are affected by these mutations. Moreover, both *AXIN2* and *MUTYH* are expressed in N14-77 samples at a level resembling other canine intestinal normal and tumor samples (Supplementary Table 2D). These observations indicate that the identified promoter mutations are unlikely pathogenic. Other genes are also expressed in N14-77 polyps and/or normal sample at a comparable level as in other canine samples (Supplementary Table 2D), indicating that germline epigenetic silencing or activating is unlikely.

### Germline mutations of other genes were identified in N14-77

#### STAMBPL1 G406D is the most notable germline missense mutation

**Figure 3:**
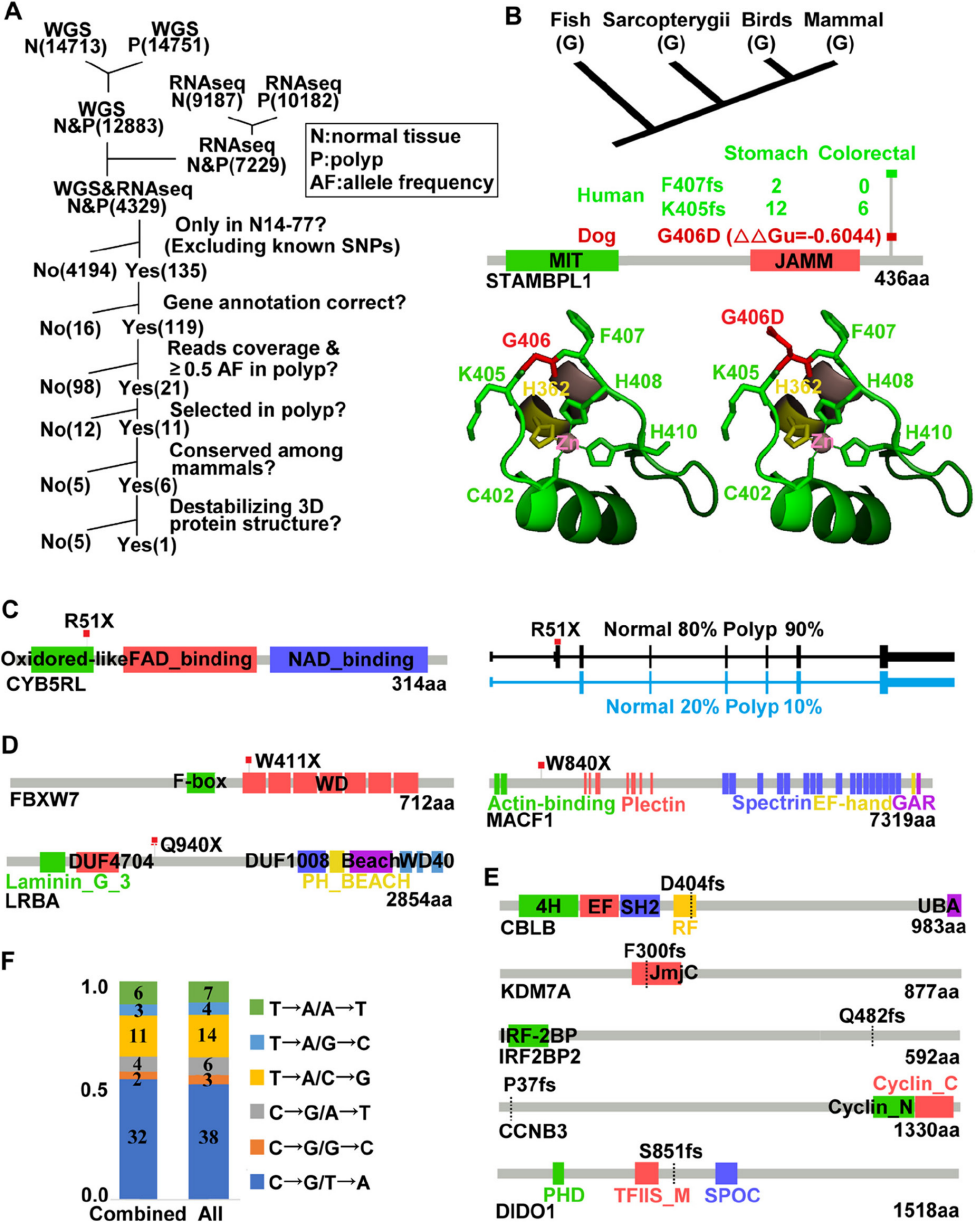
Notable germline and somatic mutations were identified in N14-77 **(A)** Outlined is our pipeline for putative pathogenic germline missense mutation discovery (see text). Numbers in parentheses indicate mutation counts. **(B)** G406D of STAMBPL1 is identified by the pipeline in A. The top image shows G406 conservation. Middle images (from bottom to top) indicate the protein domains, ∆∆Gu (red) from modeling [24] predicting that G406D likely destabilizes the protein structure, and frameshift (fs) mutations found in human cancers (with total case numbers indicated). Bottom images are the crystal structure [25] with G406 (left) and G406D (right) in red and its flanking K405 and F407 in green, and zinc-coordinating residues C402, H362, H408 and H410 shown. **(C)** R51X of CYB5RL is a heterozygous germline truncation mutation. The right image indicates the two alternative splicing forms and their proportions in each sample. **(D** and **E)** Somatic truncation mutations and frameshift mutations identified. **(F)** C→T/G→A changes dominate. Combined: somatic missense mutations identified by combining WGS and RNA-seq reads. All: those found by WGS alone, RNA-seq alone and combined. The numbers inside the bars specify the mutation counts.

After individual gene study described above, we attempted genome-wide search. GATK identified a large number of missense mutations, and we developed a pipeline (Figure 3A) to reduce false positives and to prioritize mutations. First, we selected mutations that were found in both normal and polyp samples and by both WGS and RNA-seq analyses. This step, ensuring that missense mutations of interest are indeed expressed, yields 4,329 mutations in total (Figure 3A). Second, we chose mutations that are unique to N14-77, when compared to >50 cases of sporadic canine intestinal (Supplementary Table 1C) and other cancers [6, 7], and by excluding canine SNPs from published studies [1, 23] and databases. This is because N14-77 is the only case known to have extreme intestinal polyposis. A total of 135 mutations remain after this step (Figure 3A). Third, we excluded mutations located in genes with annotation issues (e.g., retrogenes or pseudogenes). To further increase the accuracy, we selected mutations with: 1) a ≥10 WGS read coverage in both normal and polyp samples; 2) a ≥30X RNA-seq read coverage in either the normal or polyp sample; and 3) a ≥0.5 variant allele frequency in the polyp sample for either WGS or RNA-seq. These selections reduce the total mutations to 21 (Supplementary Table 3A). For heterozygous mutations, we further prioritized those being selected in polyps, i.e. with a higher mutation rate in the polyp than in the normal sample (Figure 3; Supplementary Table 3A). Lastly, we considered evolutionary conservation and excluded mutations occurring during evolution (Supplementary Table 3A). In addition, we prioritized mutations predicted to alter the protein 3D structure with modeling [24] (Supplementary Table 3A).

After processing through our pipeline (Figure 3A), G406D of STAMBPL1 is the only germline missense mutation that remains. This mutation is unique to N14-77 and being selected in polyps, with the mutation rate increasing from 67% in the normal sample to 82% in the polyp sample for WGS and from 50% to 70% for RNA-seq (Supplementary Table 3A). Furthermore, G406, the glycine residue at position 406, is conserved from fish to mammals for 100 species examined (Figure 3B). Based on modeling [24], the G406D change will likely destabilize the protein (Figure 3B).

STAMBPL1, also known as AMSH-LP, is a deubiquitinase (DUB) that cleaves K63-linked polyubiquitin chains. The crystal structure of human STAMBPL1 is determined [25]. As canine STAMBPL1 is highly homologous to its human counterpart, with the same length and sharing 92% similarity and 88% identity in amino acid sequence (Supplementary Figure 4A), we used the human structure to study the G406D mutation. STAMBPL1 is a zinc protease and contains two zinc centers. G406 locates in the 2^nd^ zinc-center, neighboring the zinc-coordinating residues C402, H408 and H410 within a highly conserved peptide (C^402^KKK^405^G^406^F^407^H^408^PH^410^) (Supplementary Figure 4B). This peptide forms a long loop, assisting the recognition and correct binding of the proximal ubiquitin of K63-linked ubiquitin chains [25]. Importantly, K405 and F407, which flank G406, are the most frequently mutated residues of STAMBPL1 in human cancers, with frameshift mutations recurrently found (Figure 3B; Supplementary Table 3B). Based on these findings, the G406D germline mutation of STAMBPL1 may be pathogenic.

#### CYB5RL harbors a germline truncation mutation

We followed the same procedure of Figure 3A, except skipping the steps of evolutionary conservation and protein 3D structure, for germline truncation mutation discovery. We manually confirmed the results with IGV and the UCSC and Ensembl genome browsers. With these, we detected a truncation mutation, R51X, in CYB5RL (cytochrome b5 reductase like) (Figure 3C). This mutation is selected in the polyps, although the allele frequency is rather low (25%) based on RNA-seq reads (Supplementary Table 3C). There appears to be a second, but minor, alternative splicing form that is not affected by this mutation (Figure 3C).

#### No germline frameshift mutations found

We applied the same strategy described above, and found no convincing germline frameshift mutations in N14-77. A small number of indels were detected, which however locate in intron regions, retrogenes, or misannotated genes based on manual examination.

### Somatic mutations were identified in N14-77 polyps

#### Somatic truncation mutations of FBXW7, LRBA and MACF1 found

We detected 7 total somatic truncation mutations in N14-77, three of which are supported by both WGS and RNA-seq analyses (Supplementary Table 3D). The 1^st^ mutation is W411X of FBXW7, occurring at a rate of 39% for WGS and 59% for RNA-seq (Figure 3D; Supplementary Table 3D). *FBXW7* is one of the most frequently (∼20%) mutated genes in human CRC [26]. The 2^nd^ mutation is Q940X of LRBA (Figure 3D), at a rate of 90% for WGS and 38% for RNA-seq (Supplementary Table 3D). LRBA (lipopolysaccharide-responsive vesicle trafficking, beach- and anchor-containing) is linked to trafficking of immune molecules such as CTLA4. LRBA deficiency, a rare genetic disorder, is associated with autoimmunity, chronic diarrhea, and B-cell deficiency [27]. The 3^rd^ mutation is W840X of MACF1 (microtubule-actin crosslinking factor 1) (Supplementary Table 3D), the loss of which disrupts epithelial cell polarity [28].

#### Somatic frameshift mutations of *CBLB* and other genes found

*CBLB* encodes an E3 ubiquitin ligase CBL-B, an immune response regulator [29]. We detected a somatic base T deletion at a rate of 50% for WGS and 29% for RNA-seq, resulting in a frameshift mutation at residue D404 (D404fs) of CBLB (Figure 3E; Supplementary Table 3D). Frameshift indels were also uncovered in *DIDO1*, involved in apoptosis [30], as well as within homopolymer sites (e.g., GGGGGG) of *KDM7A*, *IRF2BP2* and *CCNB3* (Figure 3E; Supplementary Table 3D).

#### G→A/C→T changes dominate among somatic base substitutions

We identified 72 missense mutations in total (Supplementary Table 3E). Consistent with human studies [26], G→A/C→T changes dominate over other base substitutions (Figure 3F), indicating that C/G deamination is the major somatic mutation mechanism in N14-77 polyps. Among 72 mutations, only 7 were detected by both WGS and RNA-seq analyses (Supplementary Table 3E). Furthermore, quite a few mutations could be passengers, based on evolutionary conservation and molecular modeling (Supplementary Table 3E), as well as comparison to human mutation findings (Supplementary Figure 5A). However, more studies are required to determine their driver-passenger role.

#### Somatic whole chromosome gains detected

Our analysis (Supplementary Figure 1B) revealed no translocations or inversions in the N14-77 polyp or normal genome. Neither did we find focal amplifications/deletions. We did, however, detect whole chromosome gain of chromosomes 4, 7-10, 13, 15, 23, and 26. These changes are clearly somatic, because they were only found in the polyp genome but not in the normal genome (Supplementary Figure 5B).

### Highly- and lowly expressed genes in N14-77 polyps are enriched in specific functions

**Figure 4:**
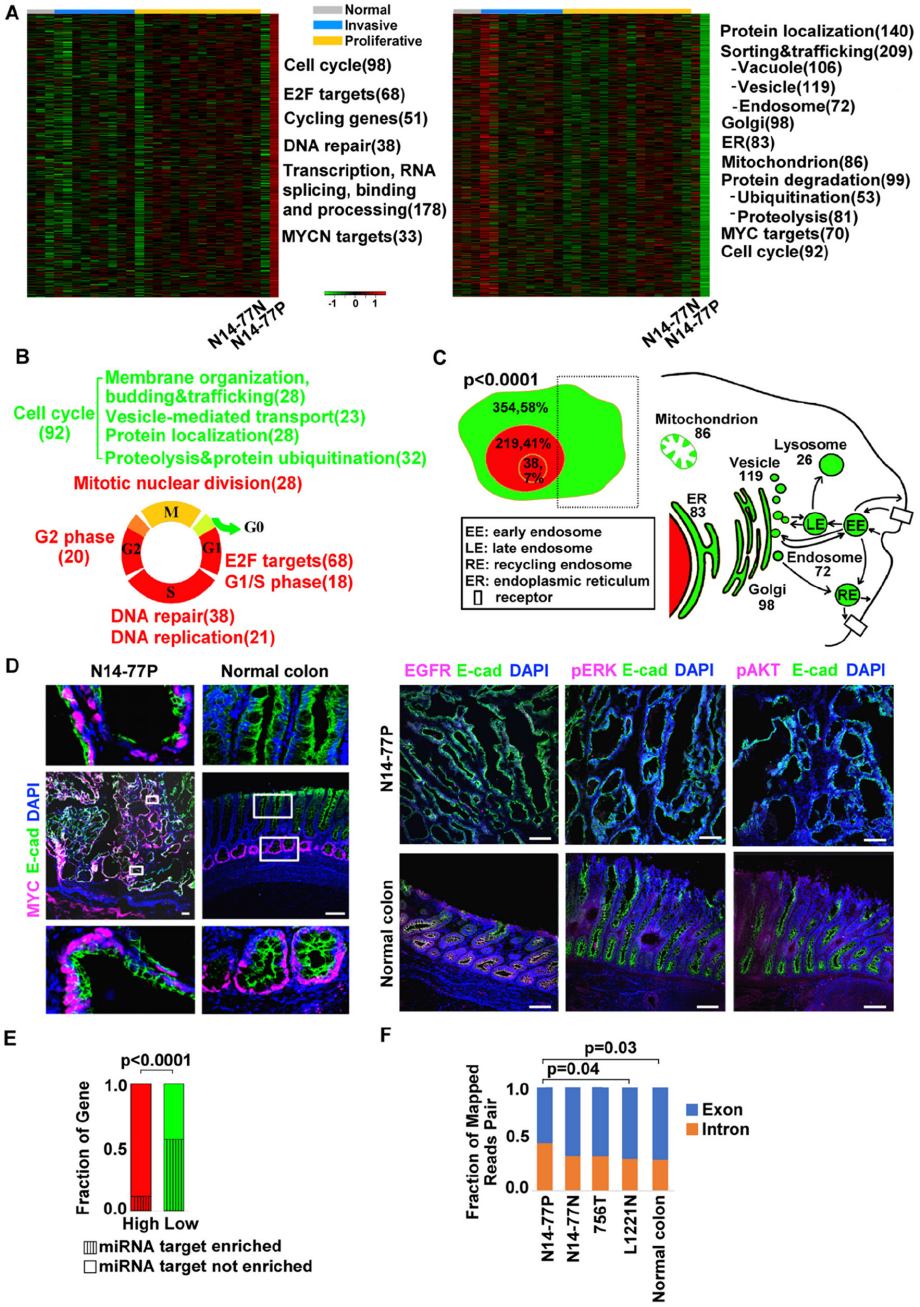
Highly- and lowly expressed genes in N14-77 polyps differ in function, cell cycle phase, cellular location and miRNA target site enrichment **(A)** Heatmaps from left to right indicate the log_2_ (*FPKM*) values of 474 highly- and 614 lowly expressed genes in 28 canine intestinal samples grouped as shown. Significantly enriched functions are listed next to the heatmap, with the total number of genes involved shown in the parenthesis. **(B)** Red and green respectively designate highly and lowly expressed genes, along with their enriched functions and cell cycle phases. Yellow indicates cell cycle phases enriched in both highly and lowly expressed genes. **(C)** Highly expressed genes are enriched in the nucleus (red) and lowly expressed genes are enriched in the cytoplasm (green), e.g., “354, 58%” indicating 354 genes, which make up 58% of all lowly expressed genes, located in the cytoplasm. The small red circle inside the nucleus designates the nucleolus. The right image illustrates that the sub cellular locations of lowly expressed genes, e.g., 83 genes are associated with ER. **(D)** Representative IHC images illustrate the enrichment of nuclear protein MYC, and the depletion of membrane and cytoplasmic proteins EGFR, pERK and pAKT, in N14-77 polyps. Scale bar, 100 μm. **(E)** Highly and lowly expressed genes differ in enriched miRNA target sites. **(F)** More RNA-seq reads were mapped to intronic regions in N14-77 polyps. 756T is a canine jejunum tumor and others are described in Figure 2E.

With our RNA-seq data from 28 samples of canine intestinal tumor and normal tissues (Supplementary Tables 1B and 1C), we identified genes that are highly or lowly expressed in N14-77 polyps. These are defined as genes with an expression level outside the expression mean ± one standard deviation range and being the highest or lowest among the 28 samples. A total of 528 highly expressed genes were identified, of which about 474 (90%) encode proteins (Figure 4A; Supplementary Table 4A). These genes are significantly enriched in functions of cell cycle, DNA repair, as well as transcription, mRNA processing and splicing (Figure 4A; Supplementary Table 4A). Meanwhile, 621 lowly expressed genes were discovered, of which only one appears to be noncoding and 614 encode proteins with functional annotation (Figure 4A; Supplementary Table 4A). Among them, the prominently enriched functions include protein localization, trafficking and degradation, as well as cell cycle.

#### >50 ubiquitin-related genes are lowly expressed

Ubiquitin-related genes are enriched only among lowly expressed genes of N14-77 polyps (Figure 4A). Specifically, a total of 53 such genes are lowly expressed (Supplementary Figure 6A), of which ≥29 are associated with ubiquitin ligases and ≥4 are linked with DUBs (Supplementary Table 4A). About 35 genes are associated with protein degradation, including 6 encoding F-box proteins (Supplementary Table 4A). Interestingly, 39 genes (73%) are enriched in microRNA (miRNA) target sites.

#### Highly and lowly expressed cell cycle genes differ in cell cycle phase and function

Although cell cycle genes are enriched in both highly and lowly expressed gene sets (Figure 4A), they differ. Highly expressed ones consist of E2F targets, and cycling genes with their expression peaking during the G1/S or G2 phase (Figure 4B; Supplementary Table 4B). DNA replication and/or repair genes, which primarily function in the S phase, and mitotic nuclear division genes are also among highly expressed (Figure 4B; Supplementary Table 4B). In contrast, 63 of the 92 lowly expressed cell cycle genes are associated with membrane organization, budding and trafficking; vesicle-mediated transport; protein localization, as well as ubiquitination and proteolysis (Figure 4B; Supplementary Table 4B). Furthermore, at least 12 lowly expressed genes encode cell cycle inhibitors, including *RB1* and *TSG101* (Supplementary Table 4B).

#### Highly and low expressed genes are enriched in different cellular locations

Approximately 41% of the highly expressed genes are annotated to be located in the nucleus (Figure 4C; Supplementary Table 4C). These include those (7%) located in the nucleolus, as well as genes functioning in DNA repair and/or replication, transcription and chromatin. This is significantly higher, when compared to both the lowly expressed genes (about 12%) and the entire gene set encoded in the genome (<25%). To the contrary, about 58% of the downregulated genes are located in the cytoplasm, associated with endosomes and other organelles (Figure 4C; Supplementary Table 4C). This is also significantly higher when compared to the highly expressed genes (∼18%) and the entire gene set encoded in the genome (<40%).

Consistent with mRNA expression (Figure 4C), our immunohistochemistry (IHC) analysis reveals depletion of EGFR, a membrane protein, and of phosphorylated ERK and AKT, both cytoplasmic proteins, in N14-77 polyps, when compared to normal intestinal tissue samples (Figure 4D). This differs from MYC, a nuclear protein (Figure 4D), as described later.

#### Lowly expressed genes are enriched in miRNA target sites

About 56% (342 genes) of lowly expressed genes are enriched in putative miRNA target sites, compared to only 16% (77 genes) for highly expressed genes (Figure 4E). Interestingly, more noncoding RNA genes are found among highly expressed genes than among lowly expressed genes, as previously described. This is consistent with that more RNA-seq reads were mapped into intronic regions in the N14-77 polyp sample, compared to the other samples (Figure 4F; Supplementary Table 4D).

#### MYC network is activated in N14-77 polyps

Several analyses indicate that the MYC network is activated in N14-77 polyps. First, the MYC protein is expressed highly and more or less uniformly throughout the polyps, ranging from the bottom to the top of the intestinal mucosa (Figure 4D). This differs from normal intestinal tissues of other dogs where MYC is only expressed at the bottom layer of the mucosa (Figure 4D). Second, MYC has the highest mRNA expression level in N14-77 polyps, among 28 canine intestinal tumor and normal samples investigated (Supplementary Figure 6B).

**Figure 5:**
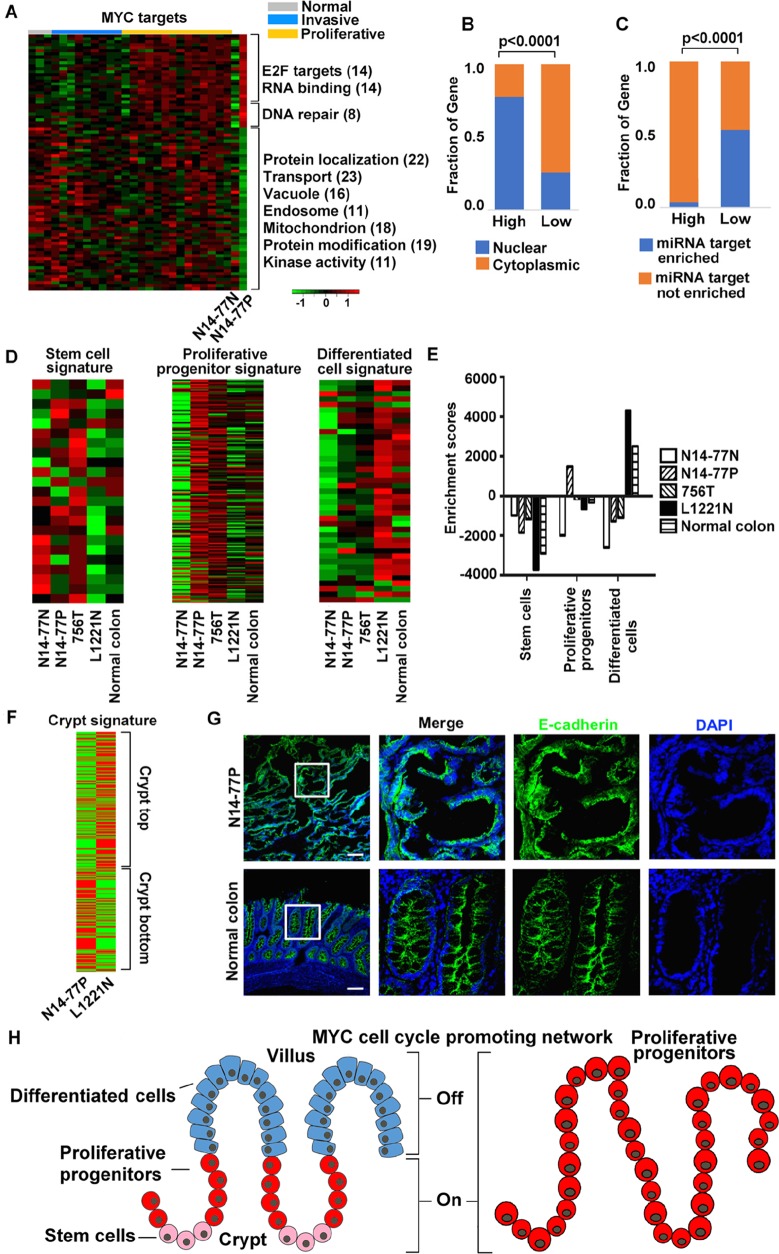
MYC network and crypt proliferative progenitor signature are activated in N14-77 polyps **(A)** MYC targets are enriched in both highly and lowly expressed genes. The image is presented as described for Figure 4A. **(B** and **C)** Highly and lowly expressed MYC target genes differ in enriched cellular locations and miRNA target sites. **(D** and **E)** Signature of intestinal proliferative progenitors, but not of either intestinal stem cells or differentiated cells, is activated in N14-77 polyps. The heatmaps indicate the log_2_ (*FPKM* values of signature genes [31] (D), and the bar plot indicate the corresponding ssGSEA results (E). **(F)** Signature [32] of the crypt bottom, but not of the crypt top, is activated in N14-77 polyps. **(G)** Representative IHC images indicate the lack of well-established epithelial apical-basolateral cell polarity in N14-77-polyp cells. Scale bar, 100 μm. **(H)** Cartoons illustrate the differentiation of normal intestinal epithelium (left) and indicate that N14-77 polyp cells are in the proliferative progenitor state (right).

Notably, MYC targets are enriched in both highly and lowly expressed gene sets of N14-77 polyps (Figure 4A). Specifically, 21 MYC targets, 38% of which function in DNA repair, and 33 MYCN targets, ∼70% of which are E2F targets and/or associated with RNA-binding and processing, are highly expressed (Figure 5A; Supplementary Table 5A). Meanwhile, a total of 70 MYC targets are lowly expressed and, except for protein degradation, are enriched in the same functions as the entire lowly expressed gene set (Figure 5A; Supplementary Table 5A). Also like the entire gene sets, highly expressed MYC targets are enriched in the nucleus (76%) and depleted in miRNA target sites (4%), while lowly expressed ones are enriched in the cytoplasm (64%) and miRNA target sites (53%) (Figure 5B and 5C).

#### N14-77 polyps exhibit crypt proliferative progenitor signature

We investigated published gene signatures that mark different intestinal epithelial differentiation stages [31] via single sample gene set enrichment analysis (ssGSEA). Signature genes of crypt proliferative progenitors, but not of either intestinal stem cells or differentiated epithelial cells, are significantly upregulated in N14-77 polyps (Figures 5D and 5E; Supplementary Tables 5D and 5E). This agrees with that N14-77 polyps display upregulated signature of the crypt bottom and downregulated signature of the crypt top [32] (Figure 5F; Supplementary Table 5F). Furthermore, also consistent with the ssGSEA results, our IHC analysis reveals that N14-77 polyp cells lack well-established apical-basolateral polarity, unlike fully differentiated epithelial cells (Figure 5G; Supplementary Figure 6D). These observations indicate that N14-77 polyp cells are in the proliferative progenitor state (Figure 5H).

### N14-77 intestinal microbiota is enriched in bacteroidetes

As described previously, medical examination indicates extensive bacterial infection in the N14-77 intestine. To better understand this, we utilized WGS and RNA-seq data to examine the intestinal microbiota. Briefly, we first identified WGS and RNA-seq read pairs of which neither read could be mapped onto the canine reference genome (Supplementary Tables 1A and 1B), which were then searched against three microbial databases. The 1^st^ database is the reference genomes curated by the Human Microbiome Project [33], referred to as HMP hereafter. The 2^nd^ database contains all bacterial genomic sequences (ABG) downloaded from the NCBI. The 3^rd^ database is simplified from ABG, consisting of genomic sequence of the longest strain of each bacterial species with genome sequencing completed. It is hence named longest bacterial genomes (LBG). We noted that the results with LBG are somewhat skewed. We thus only focus on HMP and ABG studies, as described below.

**Figure 6:**
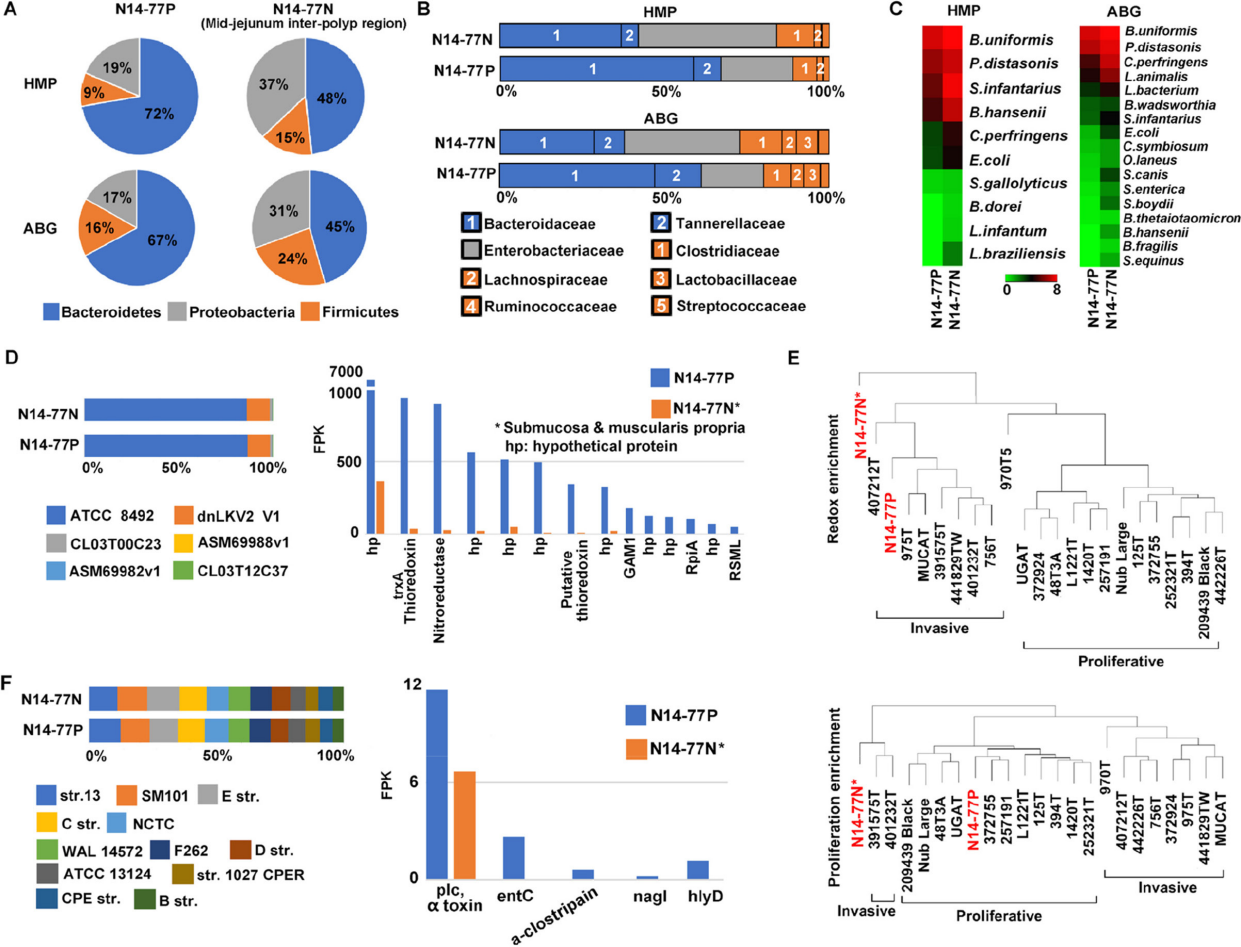
Bacteroidetes, *B. uniformis* and *C. perfringens* are significantly enriched in N14-77 intestinal microbiota **(A)** Bacteroidetes is the dominating phylum. The pie charts indicate the composition of bacterial phyla, determined by searching WGS reads against microbial genome databases HMP and ABG. **(B)** Bacteriotecea is the dominant family in polyps. The colors represent bacterial phyla as shown in A. **(C)** *B. uniformis* and *C. perfringens* are among the top enriched species. **(D)** ATCC 8492 is the most enriched strain of *B. uniformis* (left), determined with WGS reads, and expresses abundantly thioredoxin and nitroreductase genes (right), determined with RNA-seq reads. **(E)** N14-77 polyps resemble invasive tumors, but not proliferative tumors, in redox gene expression. The images indicate sample clustering based on the ssGSEA enrichment scores with indicated gene sets. **(F)** *C. perfringens* strain enrichment and toxin gene expression.

Our analysis with WGS data reveals that N14-77 samples contain more bacteria than other intestinal tumor and normal samples which we investigated (Supplementary Table 1C). More importantly, bacteroidetes is the most enriched bacterial phylum, accounting for 67-72% for polyps and 45-48% for the normal sample, followed by proteobacteria and firmicutes (Figure 6A; Supplementary Table 6A). Other phyla each makes up < 1% (Supplementary Table 6A). Our results differ from typical microbiota of canine jejunum published [34], where bacteroidetes are less enriched than proteobacteria, firmicutes, actinobacteria and spirochaetes. Instead, with bacteroidetes predominating, N14-77 samples, which are from jejunum (Figure 1), better resemble colon in microbiota [34]. This is confirmed at the family level, where bacteroidaceae, enterobacteriaceae, clostridiaceae and tannerellaceae dominate (Figure 6B; Supplementary Table 6B). Again, families of bacteroidetes, i.e. bacteroidaceae and tannerellaceae, are significantly enriched, particularly in the polyp sample. At the species level, the top enriched include *Bacteroides uniformis* and *Clostridium perfringens* (Figure 6C; Supplementary Table 6C). While both bacteria can be found in the intestine of healthy individuals, they are thousands times more enriched in N14-77 tissues, compared to other canine intestinal samples investigated (Supplementary Table 1C).

#### *B. uniformis* is highly enriched and expresses thioredoxin and nitroreductase abundantly

*B. uniformis* is the top enriched microbial species in both normal and polyp samples of N14-77 (Figure 6C; Supplementary Table 6C). Among its 6 strains examined, ATCC 8492 is about 7-800 times more enriched than others (Figure 6D; Supplementary Table 6D). Importantly, our RNA-seq data reveal that redox genes of *B. uniformis* are highly expressed in N14-77 polyps. Specifically, *trxA* which encodes thioredoxin, a redox protein, is the 2^nd^ most abundantly expressed gene, while a nitroreductase gene ranks the third highest expressed (Figure 6D; Supplementary Table 6D).

Although being proliferative but not invasive (Figure 1B), N14-77 polyps appear to have a tissue redox state that better resembles invasive tumors than proliferative tumors. With host redox-related gene sets (Supplementary Figure 7A and Supplementary Table 6E), the N14-77 polyp sample clusters with invasive tumors, instead of proliferative tumors (Figure 6E). With cell proliferation-related gene sets (Figure 6E; Supplementary Figure 7B) or in genome-wide expression (Supplementary Figure 7C), the opposite was observed.

#### *C. perfringens* is enriched and expresses α-toxin

*C. perfringens* is among the top few enriched species in N14-77 samples (Figure 6C), and is linked to conditions such as diarrhea and enteritis in dogs [35]. We hence examined *C. perfringens* in more depth. *C. perfringens* strains are classified into A, B, C, D and E types, based on major toxins produced [36]. There are 12 strains in our database: 3 type A, 2 type C, and one each for types B, D and E, plus 4 unclassified (Supplementary Table 6F). By counting WGS reads that are uniquely mapped to each strain, we note that strains 13 and SM101, both type A, and type E strain JGS1987 are slightly more enriched (Figure 6F; Supplementary Table 6F). Meanwhile, type B strain ATCC 3626 is the least enriched. Finally, we examined the expression of *C. perfringens* toxin genes with our RNA-seq data. As strain 13 represents the reference strain for *C. perfringens* and its genome is well annotated [37], we used it to identify the toxin genes and found 25 of them (except for *nanH*). In the polyp sample, we detected substantial expression of α-toxin, a phospholipase C, and an enterotoxin (*entC*), as well as trace expression of μ-toxin (*nagI*), α-clostripain and hemolysin (*hlyD*) (Figure 6F; Supplementary Table 6F).

Lastly, although medical examination suggests yeast infection, we did not find any of our WGS reads mapped to the yeast genomes in the HMP database.

## DISCUSSION

**Figure 7:**
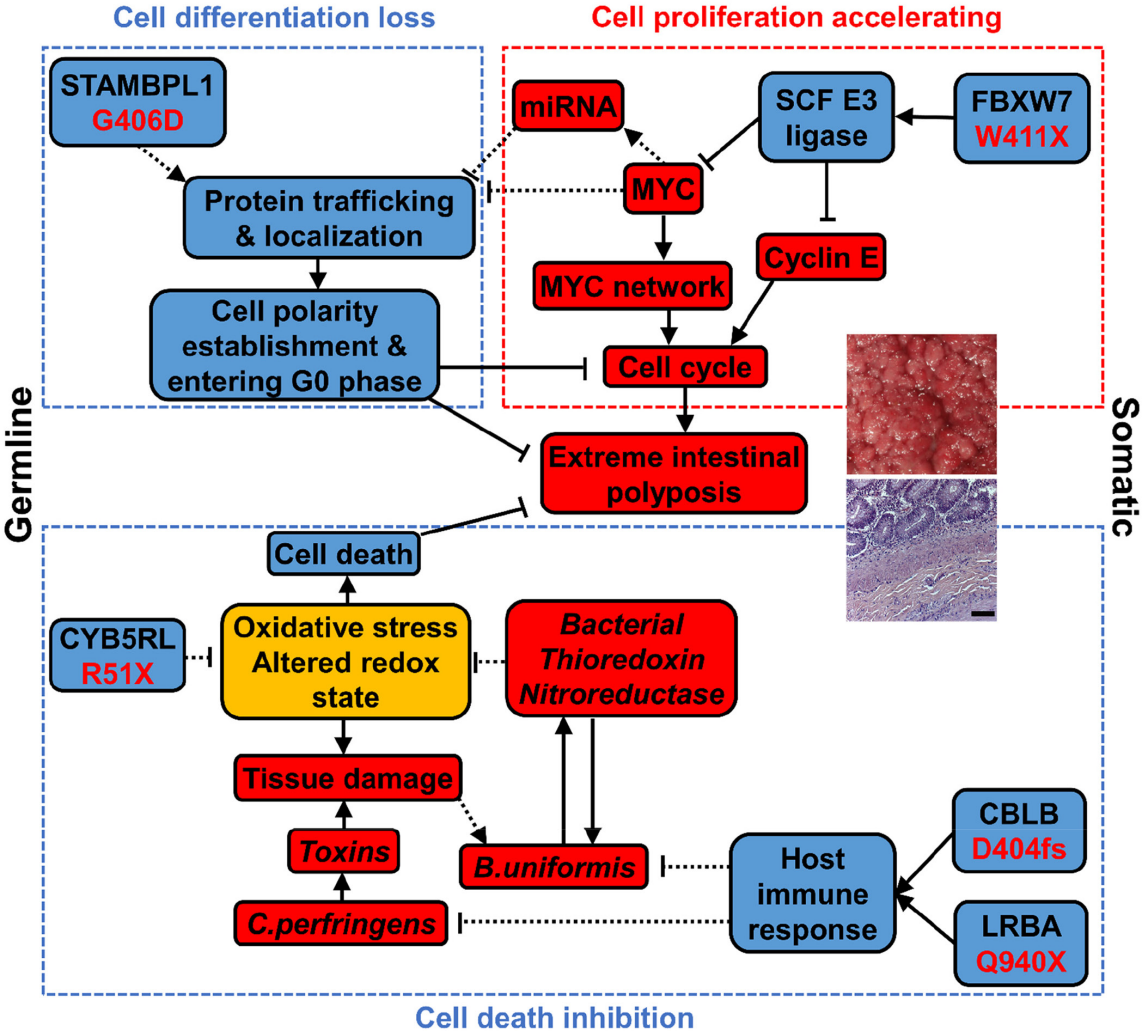
An alteration network collaborating across the genome, transcriptome and microbiome contributes to extreme intestinal polyposis of N14-77 Bacterial elements are shown in italics. Red, blue, and yellow designate promoting, inhibiting, and both promoting and inhibiting factors, respectively. “→”: promoting; “⊣”: inhibiting. Solid lines indicate that the relationship is supported by published studies and our observations, while dashed lines indicate that the relationship requires future functional validation.

N14-77 represents the first reported case of extreme intestinal polyposis in the dog. Our analysis does not support the involvement of *APC* mutations. Instead, we propose that this extraordinary phenotype is possibly caused by an alteration network collaborating across the genome, transcriptome and microbiome (Figure 7), as discussed below.

### Host ubiquitin gene alterations and MYC and cell cycle-promoting network activation keep cells proliferating

FAP and many CRCs in humans follow the pathogenic pathway of *APC* mutation → β-catenin accumulation in the nucleus → *MYC* upregulation and cell-cycle activation → cell proliferation [26]. With the lack of APC mutation, our data indicates that N14-77 has likely taken a different route: FBXW7 truncation mutation → MYC protein accumulation and cell cycle activation → cell proliferation. FBXW7, a F-box protein, constitutes the substrate-recognition subunit of the *SKP1*-*cullin*-*F*-*box (SCF)* E3 *ubiquitin* ligase that targets MYC and cyclin E for degradation [38]. The W411X truncation mutation of FBXW7 could render this SCF complex defective and unable to ubiquitinate MYC and cyclin E for degradation. Deletion of *FBXW7* in the gut has induced intestinal adenomas in mice [39].

Interestingly, 53 ubiquitin genes are downregulated in N14-77 polyps, the significance and mechanism of which clearly need further studies. Among them are genes encoding TRPC4AP and CUL4A, which constitute the MYC-targeting DDB1-CUL4 E3 ligase complex [40]. This may further lead to MYC protein accumulation.

MYC is a master transcription factor. MYC protein accumulation accelerates the transcription of numerous cell cycle promoting genes. Indeed, E2F targets, DNA repair genes, and RNA processing and slicing genes are all upregulated in N14-77 polyps. These would keep N14-77 polyp cells proliferating (Figure 7).

### Ubiquitin gene alteration and MYC network activation likely inhibit epithelial polarity establishment and cell differentiation

G406D of STAMBPL1 is the most significant germline missense mutation discovered in N14-77. STAMBPL1 (AMSH-LP) is a K63-specific DUB of the JAMM/MPN+ family [41]. G406 appears critical to its DUB activity, based on strong evolutionary conservation, crystal structure [25] and human cancer mutation findings. The G406D mutation may disrupt the DUB activity by destabilizing the 2^nd^ zinc-center, affecting substrate binding.

The function of STAMBPL1 is not well understood at present. A study indicates that it potentiates TGFβ signaling by inhibiting SMAD7 [42]. However, our ssGSEA reveals no significant difference in TGFβ signaling between N14-77 polyps and other canine intestinal tumor and normal samples (Supplementary Figure 6C). Thus, it is possible that STAMBPL1 has other functions. Its homologue STAMBP (or AMSH) is known to participate in endosomal sorting of receptors and membrane proteins [43], e.g., *STAMBP* knockdown enhancing EGFR degradation. Consistent with this, we observed depletion of EGFR, pAKT and pERK proteins in N14-77 polyp cells. Interestingly, like N14-77 polyps, stomach cancers [44] that harbor STAMBPL1 F407fs or K405fs mutation also display upregulation of MYC target genes and downregulation of trafficking genes (Supplementary Figure 4C). We propose that the DUB activity of STAMBPL1 is required for efficient sorting, trafficking and localization of proteins inside the cell. And this is disrupted by the G406D mutation, based on our model (Figure 7).

Intracellular sorting, trafficking and localization in N14-77 polyp cells are likely further disrupted by the downregulation of numerous genes associated with the system. MYC over-activation could be a contributing factor. First, many of these genes are known or putative MYC targets [45] and MYC can directly repress their transcription. Note that MYC co-repressors *ZBTB17* (*MIZ-1*) and *MXD3* are upregulated in N14-77 polyps. Alternatively, these genes could be downregulated via miRNAs, supported by that: 1) noncoding RNA genes are upregulated in N14-77 polyps; and 2) lowly expressed genes are enriched in miRNA target sites.

Intestinal epithelium develops through intestinal stem cells → proliferative progenitors → differentiated cells [31]. During the 2^nd^ stage of differentiation, the cells exit the cell cycle and establish epithelial apical-basolateral polarity. The underlying molecular mechanisms are complex. However, the intracellular sorting, trafficking and localization system clearly plays a critical role. For example, it is required to target various proteins to appropriate places to build cell adherent junctions and signaling complex (e.g., PAR, crumbs, and scribble complex) for polarity establishment [12].

Our model (Figure 7) proposes the following. Because of the STAMBPL1 G406D mutation and downregulation of genes described above, the intracellular protein sorting, trafficking and location system in N14-77 polyps is defective. This deficiency inhibits epithelial polarity establishment and cell differentiation, and prevent cells from entering the G0 phase. This, in combination with cell cycle activation, keeps N14-77 polyp cells forever in the proliferative state. Consistent with our model, N14-77 polyp cells lack well-established apical-basolateral polarity, and closely resemble intestinal proliferative progenitors.

Intestinally, our downregulated genes significantly overlap with transcripts enriched in the protruding pseudopodia formed by cells in response to migrating stimulus by fibronectin [46]. Whether this is a reason behind non-invasiveness of N14-77 polyp cells requires further investigation.

### Bacterial redox gene expression possibly reduces oxidative stress and cell death

R51X of CYB5RL is another noteworthy germline mutation uncovered in N14-77. CYB5RL is not well-studied, but its homologue cytochrome b5 reductase (CYB5R) is. The shorter soluble isoform of CYB5R is expressed in erythrocytes, catalyzing the reduction of methemoglobin (with Fe^3+^-heme) to hemoglobin (with Fe^2+^-heme). The longer isoform is expressed in other cell types. With a membrane-anchor domain, it constitutes the plasma membrane redox system which regulates the tissue redox state and reduces oxidative stress [47].

Our study reveals two isoforms of CYB5RL as well. The longer isoform is inactivated by the R51X mutation, which may cause oxidative stress, contributing to the altered tissue redox state of N14-77 polyps. Increased oxidative stress, along with bacterial toxins produced by *C. perfringens*, could result in tissue damage and lead to a faulty ileocecal valve (supported by medical examination), allowing colonic bacteria to spread to the small intestine. This may possibly explain why the N14-77 jejunum microbiota, where bacteroidetes dominate, better resembles typical microbiota of the colon rather than the jejunum. Moreover, somatic mutations of CBLB and LRBA, two key immune regulators, could alter the host immune response. We propose that, as a result of all of these, *B. uniformis* thrives (Figure 7). Importantly, *B. uniformis* bacteria express thioredoxin, especially *trxA*, and nitroreductase genes abundantly. The *trxA* gene is essential for the survival of *B. fragilis* under aerobic condition by reducing oxidative stress [48]. Like *B. fragilis, B. uniformis* is an anaerobe and normally resides in the colon, where the O_2_ level is lower than in the jejunum. Thus, we postulate that *B. uniformis* expresses thioredoxin (and nitroreductase) amply to remediate oxidative stress that is induced by the more aerobic environment of the jejunum and is exacerbated by the host CYB5RL R51X mutation. Meanwhile, host cells should also benefit. Our model proposes that by decreasing oxidative stress, these bacterial redox systems reduce host cell death and contribute to extreme polyposis (Figure 7).

In summary, we propose that three pathways lead to N14-77 extreme intestinal polyposis (Figure 7). First, MYC and cell cycle-promoting network activation, caused by a FBXW7 somatic mutation-initiated SCF E3 ubiquitin ligase defect, keeps crypt cells dividing. Second, defective intracellular trafficking and localization, originating from D406G germline mutation of STAMBPL1 and enhanced by MYC network activation, inhibit cell polarity establishment and cell differentiation, preventing cell cycle exit. Lastly, bacterial redox systems reduce the oxidative stress caused by germline mutation R51X of CYB5RL, decreasing cell death. Lastly, we emphasize that future functional studies are required to validate our model.

## MATERIALS AND METHODS

### Canine tissue samples

Fresh-frozen (FF) canine intestinal normal tissues and spontaneous tumors were obtained from various Veterinary Colleges (Supplementary Table 1C). Samples were collected from client-owned dogs that develop the disease spontaneously, under the guidelines of the Institutional Animal Care and Use Committee for use of residual diagnostic specimens and with owner informed consent. The breed, age, histopathologic descriptions, and other information are provided in Supplementary Table 1C.

### Tissue dissection, DNA and RNA extraction, and quality control

Cryosectioning of FF tissues, H&E staining and cryomicrodissection were performed as described [5, 7] to enrich polyp/tumor cells for the polyp/tumor sample, as well as unaffected/normal cells for control/normal samples. Genomic DNA and RNA were then extracted from the dissected tissues using the AllPrep DNA/RNA Mini Kit (cat. no. 80204) from QIAGEN. Only samples with a 260/280 ratio of ∼1.8 (DNA) or ∼2.0 (RNA) and showing no degradation and other contaminations were subjected to further quality control with qPCR and qRTPCR analysis with a panel of genes as previously described [7, 9].

### Paired-end WGS and RNA-seq

Both types of sequencing were conducted using the Illumina platform, following the protocols from the manufacturer. Paired-end 125 x 125bp WGS was performed in collaboration with the BGI-America and the High Throughput Genomics Core Facility at Huntsman Cancer Center at the University of Utah. RNA-seq was performed in collaboration with the Georgia Genomics Facility at the University of Georgia.

### Sequence data analyses

The overall sequence analysis pipeline was summarized in Supplementary Figure 1 and described in details in Supplementary methods. Briefly, WGS reads were aligned to the dog reference genome canFam3.1 [1] with BWA [49] v0.7.10. RNA-seq reads were mapped to the same reference genome using either TopHat [50] 2.1.1 (for gene expression) or STAR [51] v2.4.1c (for mutation finding). Three canine gene annotation databases were used, including Ensembl and the Broad annotation [22], both RNA-seq based, and human xenoRefGene [7]. Known canine SNPs used include those reported in other canine samples by us [6, 7] and the Broad Institute [1], as well as data from the NCBI, Ensembl, and DoGSD [23] databases. Both WGS and RNA-seq reads were used for germline mutation discovery with GATK [52] v3.6 and for somatic mutation finding with MuTect [53], following pipelines recommended by the Broad Institute. WGS data were used to identify germline and somatic inversions/translocations and chimeric fusion genes as described before [5–7]. For copy number changes, correctly and uniquely mapped WGS read pairs were used to calculate mapped pair density per 1kb tiling window along a chromosome. Each density was normalized against the corresponding value of a control genome and then used for germline and somatic copy number change discovery as previously described [5–7]. Gene expression quantification with RNA-seq reads and other analyses were performed as previously described [6, 7].

### Microbiome analysis

WGS and RNA-seq read pairs that could not be placed onto the canine genome were mapped with BWA v0.7.10 to three microbial genome databases – HMP, ABG and LBG. HMP is the reference genomes curated by the Human Microbiome Project [33]. HMP consists of genomic sequences of bacteria (1751 strains from 1253 species), viruses (3683 strains from 1420 species), archaea (131 strains from 97 species) and 326 lower eukaryotic species. ABG contains all bacterial genomic sequences (ABG) downloaded from the NCBI, with 2,845,483 sequences in total from 2679 species. LBG is simplified from ABG by: 1) selecting species with complete genomic sequences; and 2) for species with multiple strains having complete genomic sequences, selecting the longest strain. LBG consists of 1,576 bacterial species.

Mapped WGS read pairs were used to estimate microbial enrichment in each sample. First, the taxonomy data downloaded from the GOLD database (gold.jgi.doe.gov) were used to classify each bacterial species. Second, mapped WGS read pairs were selected as follows. For pairs with at least one read uniquely mapped, those with mapping quality Q >0 were selected. For pairs with both reads duplicatedly mapped, those that are correctly mapped (i.e., both reads mapped to the same DNA fragment, in correct orientation and spanning a reasonable genomic distance) were selected. Third, each selected read pair was assigned as follows. A read pair was assigned to a phylum and counted as one, if it was mapped to this phylum only and no matter how many times it was mapped within this phylum. If a read pair was mapped to ≥ 2 different phyla, it was discarded. Lastly, read pairs assigned to each phylum was tallied and used to estimate the phylum enrichment. The same procedure was followed to estimate the family, species and strain enrichment.

Bacterial genome annotation data were downloaded from NCBI and Ensembl. HTSeq [54] v0.6.1 was used to tally correctly and uniquely mapped RNA-seq reads pairs within each gene, which were then used to estimate the expression levels of bacterial genes.

### Immunohistochemical analysis

Immunohistochemical (IHC) experiments were performed with 5-μm tissue sections as described [7]. Primary antibodies used include including those against E-cadherin (R&D Systems, AF648), β-catenin (Santa Cruz, sc-7199), MYC (Abcam, ab32072), EGFR (BioGenex, PU335-UP), phospho-Erk1/2 (Cell Signaling Technology, #4370) and phospho-Akt (Cell Signaling Technology, #4060). Alexa Fluor®488–, 647– or 594–conjugated secondary antibodies are from Jackson ImmunoResearch. Images were taken with a Zeiss LSM 710 confocal microscope.

### Data access

Sequence data have been submitted to the NCBI SRA database with accession number PRJNA418842.

## SUPPLEMENTARY MATERIALS FIGURES AND TABLES





## Abbreviations

FAP: familial adenomatous polyposis
CRC: colorectal cancer
HNPCC: hereditary nonpolyposis colorectal cancer
WGS: whole genome sequencing
IHC: immunohistochemistry
GSEA: gene set enrichment analysis
ssGSEA: single sample gene set enrichment analysis
HMP: the reference genomes curated by the Human Microbiome Project
ABG: all bacterial genomic sequences
LBG: the longest bacterial genomic sequences
