# Supplementary material for: Collaborating genomic, transcriptomic and microbiomic alterations lead to canine extreme intestinal polyposis
Source: Oncotarget. 2018 Jun 26;9(49):29162–79. doi: 10.18632/oncotarget.25646 (PMC6044369; doi:10.18632/oncotarget.25646)
Supplement: Supplementary file 1 [file oncotarget-09-29162-s001.pdf]

Collaborating genomic, transcriptomic and microbiomic alterations lead to canine extreme intestinal polyposis

SUPPLEMENTARY MATERIALS

N14-77 CASE INFORMATION

A nine-year-old, neutered male, Golden Retriever-mix dog was presented to the Texas A&M University Veterinary Medical Teaching Hospital with a two-month history of blood-tinged, watery diarrhea. At presentation, the dog was in poor body condition (23.7 kg; body condition score 2/9) with marked wasting of all muscles. Complete blood count reveals a microcytic, hypochromic, regenerative anemia (20.8% packed cell volume) with a severe neutrophilia (73,150 cells/μL; reference 3000-11500 cells/μL) and hypoalbuminemia (1.4 g/dL; reference 2.4-3.6 g/dL). Loops of thickened, gas distended small intestine suggestive of enteritis were identified with abdominal radiographs. On abdominal ultrasound, the wall of multiple sections of jejunum is thickened up to 8 mm, and the lumen is occluded by an ill-defined mass that effaces the normal architecture of all layers of the intestinal wall. A rectal scraping reveals numerous, degenerate neutrophils containing phagocytosed bacteria and small yeast. The differential clinical diagnoses include either an infectious process or effacement by disseminated neoplasia. Euthanasia was elected based on the patient's poor clinical condition and the extent of the intestinal changes.

A full necropsy was permitted. Approximately 70% of the small intestinal mucosa, extending from the mid-jejunum to 5 cm aboral to the ileocecal junction, is severely thickened by innumerable, 3 mm to 1.1 cm, firm nodules that progressively coalesces into large, plaque-like, 10-30 cm-long areas with a red, granular surface. The duodenum and proximal jejunum orad to the affected region are diffusely congested and filled with copious mucus. No significant abnormalities were found in any of the other organ systems. Samples of the affected intestine were flash-frozen and stored at -80°C for genetic analysis. Samples of the intestine and major organs were fixed in neutral buffered formalin and embedded in paraffin (FFPE) for routine histopathologic examination with hematoxylin and eosin staining (H&E staining).

On histologic examination, numerous single to coalescing polyps are within the mucosa of the jejunum and proximal colon, and the epithelium from the crypts to mucosal surface are uniformly hyperplasitc. The mucosa comprising the inter-polyp regions and within the distal colon also have mild to moderate mucosal hyperplasia with variable neutrophilic infiltration and mild enterocolitis. Neither malignant neoplastic transformation of nor invasion of the lamina propria by enterocytes lining the intestinal villi, crypts, or colonic glands was observed.

DETAILED SEQUENCE DATA ANALYSIS

APC annotation curation

Three canine gene annotation databases were used, including Ensembl and the Broad annotation (18), both RNA-seq based, xenoRefGene, built by mapping the transcript or protein sequences of the human or other species onto the dog genome. To simplify the analysis, we selected the xenoRefGene data that use human sequences only. *APC* annotation data (transcripts, exons, introns, coding regions, etc.) were extracted from each database, and compared to those of the human *APC* from the RefSeqGene database to identify the discrepancy (Figure 2A). Genomic sequence errors in canine *APC* gene were identified by aligning both canine WGS and RNA-seq reads (Supplementary Table 1) to the canine reference genome canFam3.1 (1) using BWA (Figure 2B). These errors were verified via manual examination with IGV. After removing the identified errors, *APC* gene and transcripts were reassembled by using RNA-seq reads with Tophat and Cufflinks. The longest coding region was identified for each transcript. The translated protein sequences were then aligned with the canonical sequence of human APC protein to identify the domains and the amino acid insertions/deletions (Figure 2C).

Germline mutation finding

Both WGS and RNA-seq data of N14-77 were used for germline mutation discovery. First, WGS reads were aligned to the dog reference genome canFam3.1 (1) with BWA and locally realigned with GATK. Sorted bam files were generated by SAMtools. The duplicated mapped reads marked with Picard were excluded. Then, sequence mutation (base substitution and small indel) findings were conducted using GATK. Mutation finding with RNA-seq

reads were performed following a pipeline developed by the Broad Institute. Briefly, RNA-seq reads were mapped by STAR, realigned, and mutations were then identified by GATK.

Germline missense mutation discovery were conducted following the pipeline outlined in Figure 3A via artifact reduction and mutation prioritization. The 1st step is to identify mutations found in both normal and polyp samples and by both WGS and RNA-seq analyses. The 2nd step is to exclude known SNPs reported in other canine samples by us (Supplementary Table 1C) (5, 6), the Broad Institute (1) and others, including the SNP data at the NCBI, Ensembl, and DoGSD (19) databases. The 3rd step is to exclude mutations located in genes that are likely retrogenes or pseudogenes. The 4th step is to select only those mutations with: 1) a ≥10 WGS read coverage in both normal and polyp samples; 2) a ≥30X total RNA-seq read coverage in either the normal or polyp sample; and 3) a ≥0.5 variant allele frequency in the polyp sample for either WGS or RNA-seq. The 5th step is to select mutations with a higher mutation rate in the polyp sample than in the normal sample, i.e., being selected in the polyp sample, for heterozygous mutations. The last step is to prioritize mutations predicted to stabilize/destabilize the protein 3D structure based on modeling (20).

Germline truncation mutations and frameshift indels were discovered by identifying those that were: 1) found by both WGS and RNA-seq analyses in both polyp and normal samples; 2) unique to N14-77; 3) with a ≥10 WGS read coverage in both normal and polyp samples, a ≥30X total RNA-seq read coverage in either the normal or polyp sample, and a ≥0.5 variant allele frequency in the polyp sample for either WGS or RNA-seq; and 4) confirmed by manual examination with IGV and the UCSC and Ensembl genome browsers.

Somatic mutation finding

Both WGS and RNA-seq reads were used for somatic mutation discovery. Reads alignment to the reference genome, local realignment, and duplicatively mapped read exclusion were performed as described above. Then, MuTect was used for somatic truncation and missense mutation discovery, following the pipeline recommended by the Broad Institute. Somatic indels were identified by GATK, defined as those that were found in the polyp sample by both WGS and RNA-seq analyses, but not detected in the normal sample by either method. To reduce false positives, we excluded indels already reported in the canine SNP databases or identified in other canine samples, as described previously. Finally, mutations identified were confirmed by manual examination with IGV and the UCSC and Ensembl genome browsers.

Copy number change and structural rearrangement identification

WGS data were used to identify germline and somatic copy number changes, as well as inversions/translocations and chimeric fusion genes as previously described (4-6) and outlined in Supplementary Figure 1.

For copy number changes, correctly and uniquely mapped WGS read pairs using BWA were used to calculate mapped pair density per 1kb tiling window along a chromosome. Then, $\log_2 \frac{di}{\bar{d}}$, where d_i and $\bar{d}$ respectively represent the mapped pair density of window i and the genome-wide average of d_i, were calculated for each chromosome for each sample. Then, change points among $\log_2 \frac{(di/\bar{d})N14-77P}{(di/\bar{d})normal\ genomes}$ or $\log_2 \frac{(di/\bar{d})N14-77N}{(di/\bar{d})normal\ genomes}$ data points for germline copy number change, where "normal genomes" represent pooled WGS data from other normal canine tissues (Supplementary Table 1C) except for N14-77N, and among $\log_2 \frac{(di/\bar{d})N14-77P}{(di/\bar{d})N14-77N}$ data points for somatic copy number change, were identified following a strategy as previously described (4-6). Significantly altered segments were identified as described (4-6) by selecting those segments that are with: 1) q ≤ 0.005; 2) a size ≥ 1kb; and 3) average $\log_2$-ratio outside of mean ± standard-deviation (Supplementary Figure 1). Then, focal and broad events were identified as outlined in Supplementary Figure 1.

Translocations and inversions were identified by applying BreakDancer and CREST on WGS data. Chimeric fusion genes were uncovered by examining genes flanking the translocation breakpoints, in combination with RNA-seq data analyzed with Tophat-fusion.

Highly and lowly expressed gene identification

Gene expression quantification with RNA-seq reads and other analyses were performed as previously described (5, 6) (Supplementary Figure 1). Briefly, RNA-seq read pairs were aligned to the dog reference genome canFam3.1 with TopHat. The uniquely mapped pairs were used to

quantify a gene's expression level by calculating its FPKM value using Cufflinks with the default parameters. Three canine gene annotation databases were used, including Ensembl annotation (RNA-seq based), xenoRefGene as previously described (4-6), and our own canine annotation (also RNA-seq based). Highly or lowly expressed genes in the N14-77 polyp sample were identified as those with an expression level: 1) outside the expression mean ± one standard deviation (SD) range and 2) being the highest or lowest among the 28 canine intestinal samples investigated (Supplementary Table 1).

Gene function, cellular location, and miRNA target enrichment analysis

Gene function and other enrichment analyses were performed using GSEA (software.broadinstitute.org/gsea/index.jsp), and DAVID (david.ncifcrf.gov). Human cancer mutation data were obtained from the cBioPortal database (www.cbioportal.org).

Data and software tools used

**Deposited Data**		
WGS and RNA-seq data	This paper	SRA, SRP125500
Dog reference genome CanFam3.1	Broad Institute	http://genome.ucsc.edu/cgi-bin/hgGateway?db=canFam3
Genomes OnLine Database (GOLD)	JGI	https://gold.jgi.doe.gov/
NIH Human Microbiome Project (HMP)	HMP	https://hmpdacc.org/
All bacteria database (ABG)	NCBI	ftp://ftp.ncbi.nih.gov/genomes
Human cancer RNA-seq data	The Cancer Genome Atlas (TCGA)	https://portal.gdc.cancer.gov/
Human cancer mutation data	cBioPortal	http://www.cbioportal.org/
Canine gene annotation data	Broad Institute; EMBL-EBI; UCSC	http://genome.ucsc.edu/cgi-bin/hgGateway?db=canFam3
Canine SNP data	Broad Institute	ftp://ftp.broadinstitute.org/distribution/assemblies/mammals/dog/
Canine SNP data	Ensembl	ftp://ftp.ensembl.org/pub/release-91/variation/gvf/canis_familiaris/
Canine SNP data	DoGSD	http://dogsd.big.ac.cn/
Canine SNP data	NCBI	ftp://ftp.ncbi.nih.gov/snp/organisms/dog_9615/
**Software and Algorithms**		
BWA v0.7.10	Li and Durbin, 2009	http://bio-bwa.sourceforge.net/
Cufflinks v2.2.1	Trapnell et al., 2011	http://cole-trapnell-lab.github.io/cufflinks
TopHat 2.1.1	Trapnell et al., 2009	http://ccb.jhu.edu/software/tophat/index.shtml
ssGSEA	Barbie et al., 2009	http://software.broadinstitute.org/cancer/software/genepattern/modules/docs/ssGSEAProjection/4
STAR v2.4.1c	Dobin et al., 2013	https://github.com/alexdobin/STAR
Picard v1.60	NA	https://github.com/broadinstitute/picard
DAVID v6.8	Jiao et al., 2012	https://david.ncifcrf.gov/

GSEA v5.2	Subramanian et al., 2005	http://software.broadinstitute.org/gsea/index.jsp
GATK v3.6	McKenna et al., 2010	https://software.broadinstitute.org/gatk/
MuTect	Cibulskis, K. et al., 2013	http://archive.broadinstitute.org/cancer/cga/mutect
EASE-MM	Folkman et al., 2016	http://sparks-lab.org/server/ease/
IGV v2.1.23	Robinson et al., 2011	http://software.broadinstitute.org/software/igv/
PyMOL v1.7.4	DeLano, 2002	https://pymol.org/2/
Coot v0.8.7	Emsley and Cowtan, 2004	https://www2.mrc-lmb.cam.ac.uk/personal/pemsley/coot/
Clustal Omega (EBI web server)	Sievers et al., 2011	http://www.clustal.org/omega/
BreakDancer v1.1.2	Chen et al., 2009	http://breakdancer.sourceforge.net/
CREST v1.0	Wang et al., 2011	http://www.stjuderesearch.org/site/lab/zhang
HTSeq v0.6.1	Anders, et al., 2015	http://htseq.readthedocs.io/en/release_0.9.1/
ANNOVAR v2015Mar22	Wang, et al., 2010	http://annovar.openbioinformatics.org/en/latest/
SEG	Tang. et al. 2010	https://www.ncbi.nlm.nih.gov/pmc/articles/PMC2840980/
R v3.3.2	RC Team, 2000	https://www.r-project.org/
Samtools v1.2	Li et al., 2009	http://samtools.sourceforge.net/

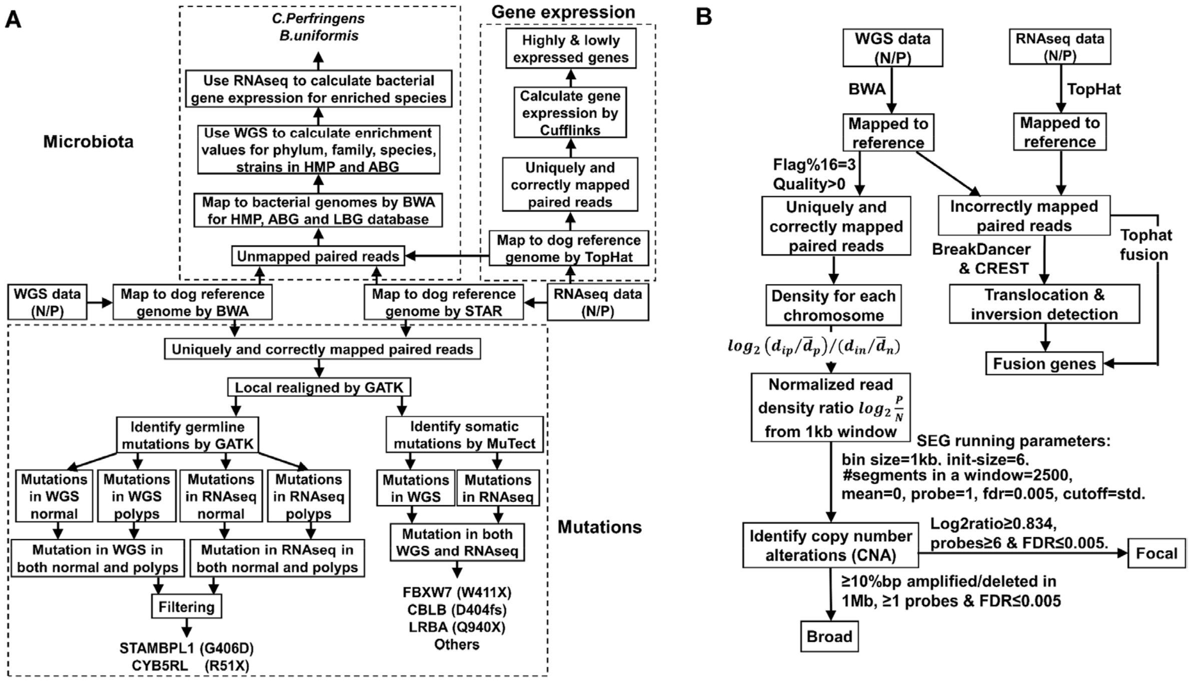

Supplementary Figure 1: Data analysis pipeline, Related to Figure 1. **(A)** Analysis pipeline of N14-77 WGS and RNA-seq data for mutation, transcriptomic and microbiomic alteration discovery. **(B)** Analysis pipeline for copy number alteration and genomic rearrangement identification.

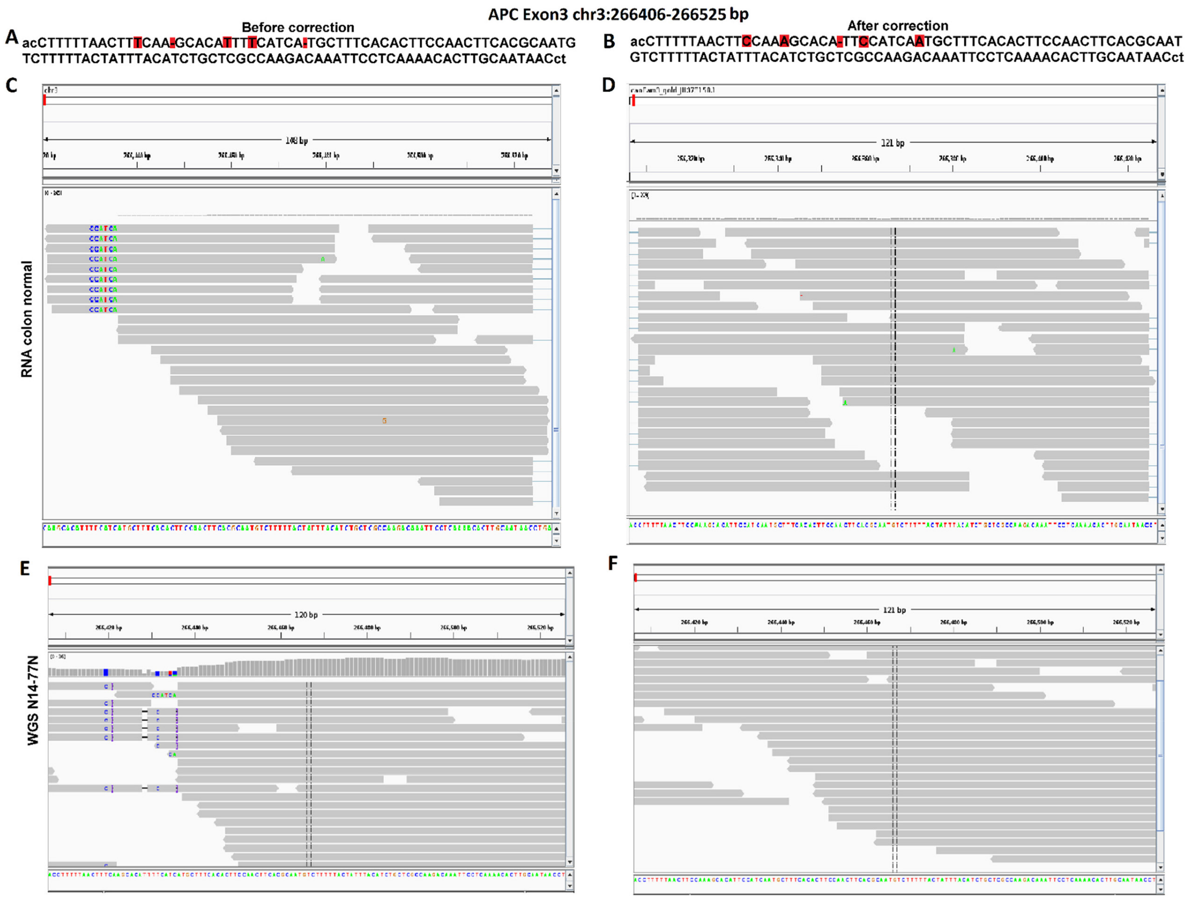

Supplementary Figure 2: Neither germline nor somatic mutations of APC were found in N14-77, Related to Figure 2. **(A** and **B)** Genomic sequences of *APC* exon3 of canFam3.1 before and after correction of the 5 sequence errors highlighted in red, including two base substitutions, two base deletions and one base insertion. Uppercase letters indicate exonic sequence, while lowercase letters indicate intronic sequence. **(C** and **D)** Captured IGV images show the alignment of canine RNA-seq reads to the corresponding genomic sequence indicated in A and B. **(E** and **F)** Captured IGV images show the alignment of WGS reads to the corresponding genomic sequence indicated in A and B.

A

Hotspots mutation

Position	WT	Noraml	N14-77N (RNA)	N14-77P (RNA)	N14-77N (WGS)	N14-77P (WGS)
257322	G	20/20	76/76	24/24	14/14	13/13
257323	C	19/19	76/76	25/25	13/13	13/13
257324	A	19/19	76/76	25/25	13/13	13/13
257325	T	19/19	76/76	25/25	13/13	13/13
257326	G	19/19	77/77	25/25	13/13	13/13
257688	T	27/27	71/71	43/43	12/12	14/14
257689	T	27/27	71/71	43/43	12/12	14/14
257690	T	27/27	70/70	44/44	12/12	15/15
257691	C	27/27	70/70	47/47	12/12	15/15
257692	T	27/27	72/72	47/47	11/11	15/15
258432	G	31/31	83/83	36/36	15/15	16/16
258433	T	31/31	82/82	36/36	15/15	16/16
258434	T	30/30	84/84	36/36	15/15	16/16
258435	T	30/30	84/84	35/35	15/15	16/16
258436	T	28/28	82/82	32/32	15/15	16/16

B

N14-77N(WGS)

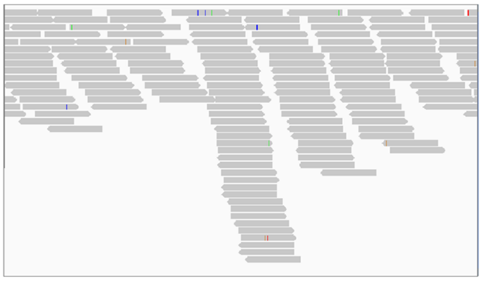

C

N14-77P(WGS)

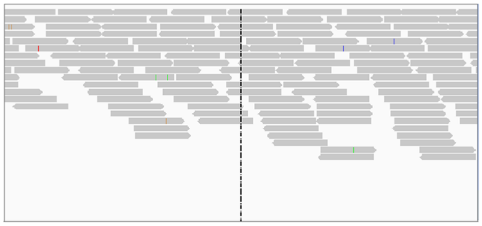

D

Normal Colon(RNA)

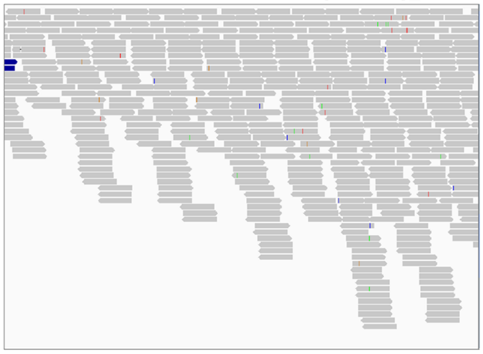

E

N14-77N(RNA)

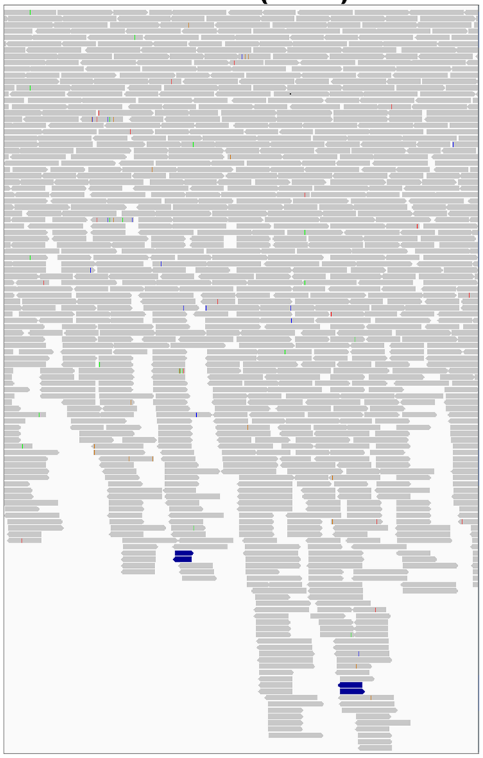

F

N14-77P(RNA)

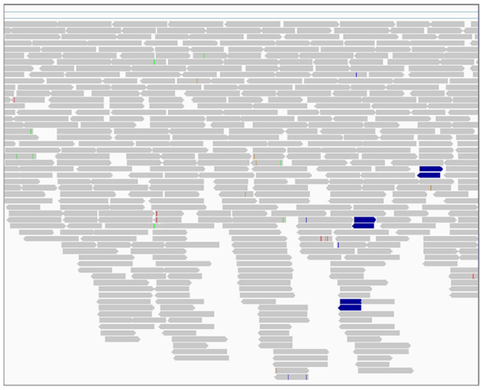

Supplementary Figure 3: Neither germline nor somatic mutations of APC were found in N14-77, Related to Figure 2. **(A)** The table indicates that no mutations were found in canine genomic sites that correspond to some of the human *APC* mutation hotpots. The 1st column shows the canine chr3 base-pair positions, and the 2nd column specifies the wildtype (WT) bases at these positions. The numbers in the remaining columns indicate the wildtype base rate of each sample at each position, e.g., 20/20 meaning that 20 out of 20 total aligned reads to the site match the wildtype base (thus no mutations). Normal: colon sample from a normal dog. **(B** and **C)** Captured IGV images show the alignment of WGS reads of N14-77 samples to the genomic sequences that span the genomic regions indicated in the Table of A (chr3:257,322-258,436bp). No convincing mutations were noted. **(D-F)** Captured IGV images show the alignment of RNA reads of N14-77 samples and the normal colon sample to the same genomic region of A and B. Again, no convincing mutations were noted.

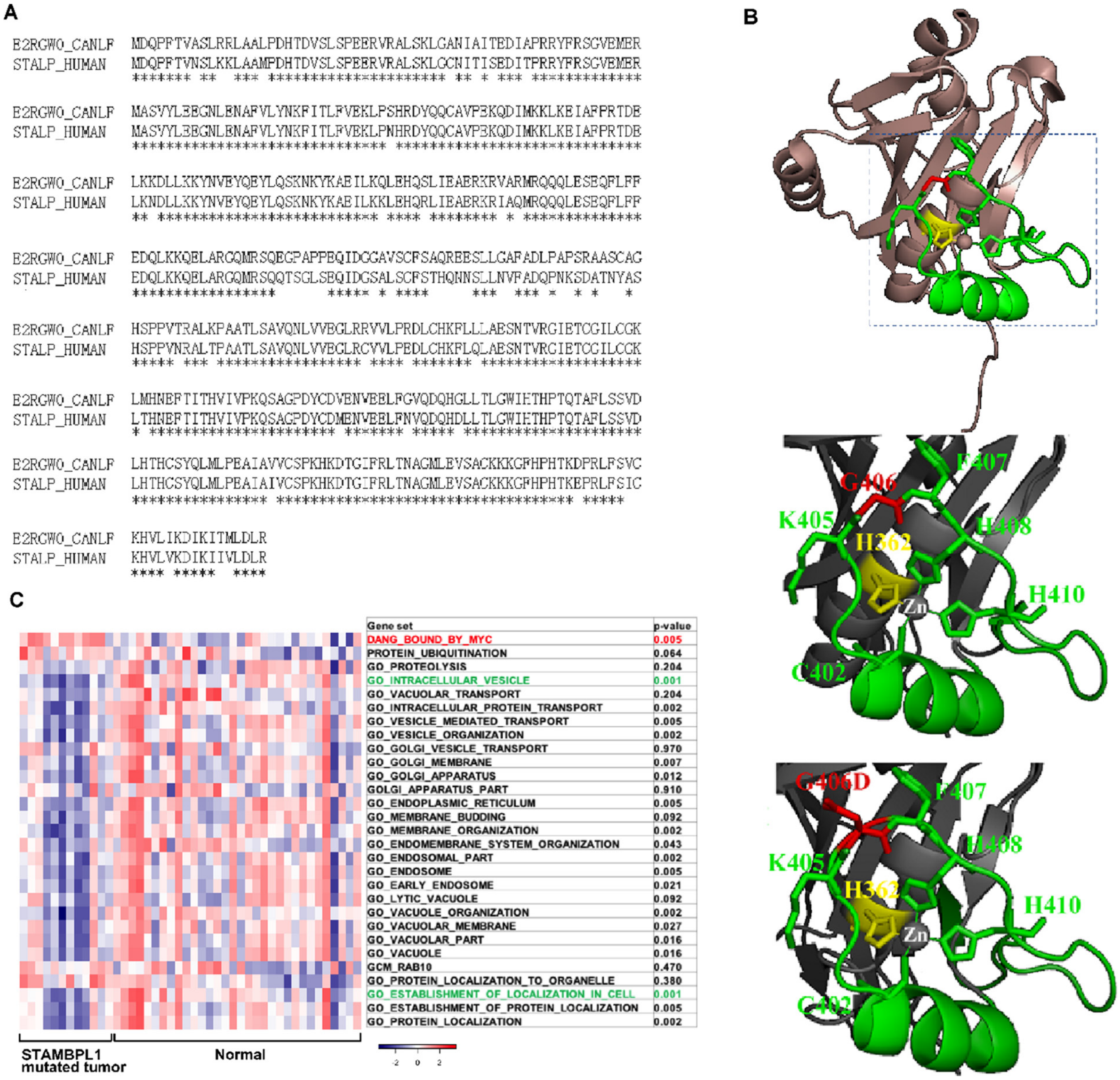

Supplementary Figure 4: G406D of STAMBPL1 is a notable germline mutation based on sequence conservation, crystal structure and human cancer mutation findings, Related to Figures 3 and 7. (A) The alignment indicates that the canine protein is highly homologous to the canonical isoform of human STAMBPL1. **(B)** Top image is the published crystal structure (2znr) of human STAMBPL1 (the canonical isoform), with the green loop consisting of residues 391-419. Images below are blowups of the portion of the structure enclosed by the square (top), showing the wildtype (middle) and the G406D mutation (bottom). **(C)** Heatmap indicates ssGSEA enrichment scores of signature genes specified in stomach cancers with STAMBPL1 F407fs or K405fs mutation (Supplementary Table 3B) and normal stomach samples from the TCGA RNA-seq study. Statistic tests indicate that vesicle-related trafficking genes are downregulated, whereas MYC related genes are upregulated, in these stomach cancers. The most upregulated and downregulated signatures are highlighted in red and green, respectively.

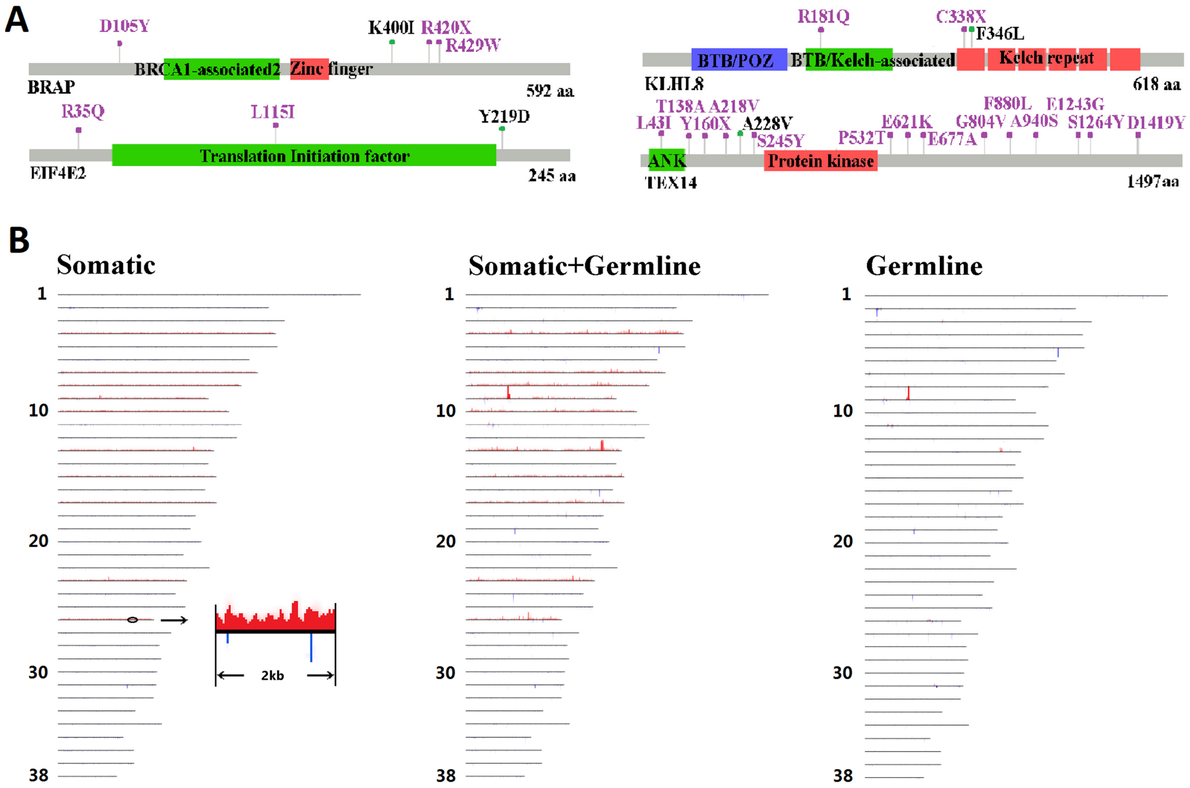

Supplementary Figure 5: Putative passenger mutations in ubiquitin genes and copy number alterations in N14-77 polyps, Related to Figure 3. (A) Dog-human comparison indicates that somatic missense mutations of the ubiquitin genes shown are likely passengers. Canine mutations are shown in black, while human mutations are shown in purple. **(B)** Copy number changes in N14-77: only somatic chromosome gains are observed. Copy number changes were identified by comparing WGS data between N14-77P and N14-77N (Somatic), between N14-77P and pooled normal genomes of canine intestinal samples (Somatic+Germline), and between N14-77N and pooled normal genomes of canine intestinal samples (Germline).

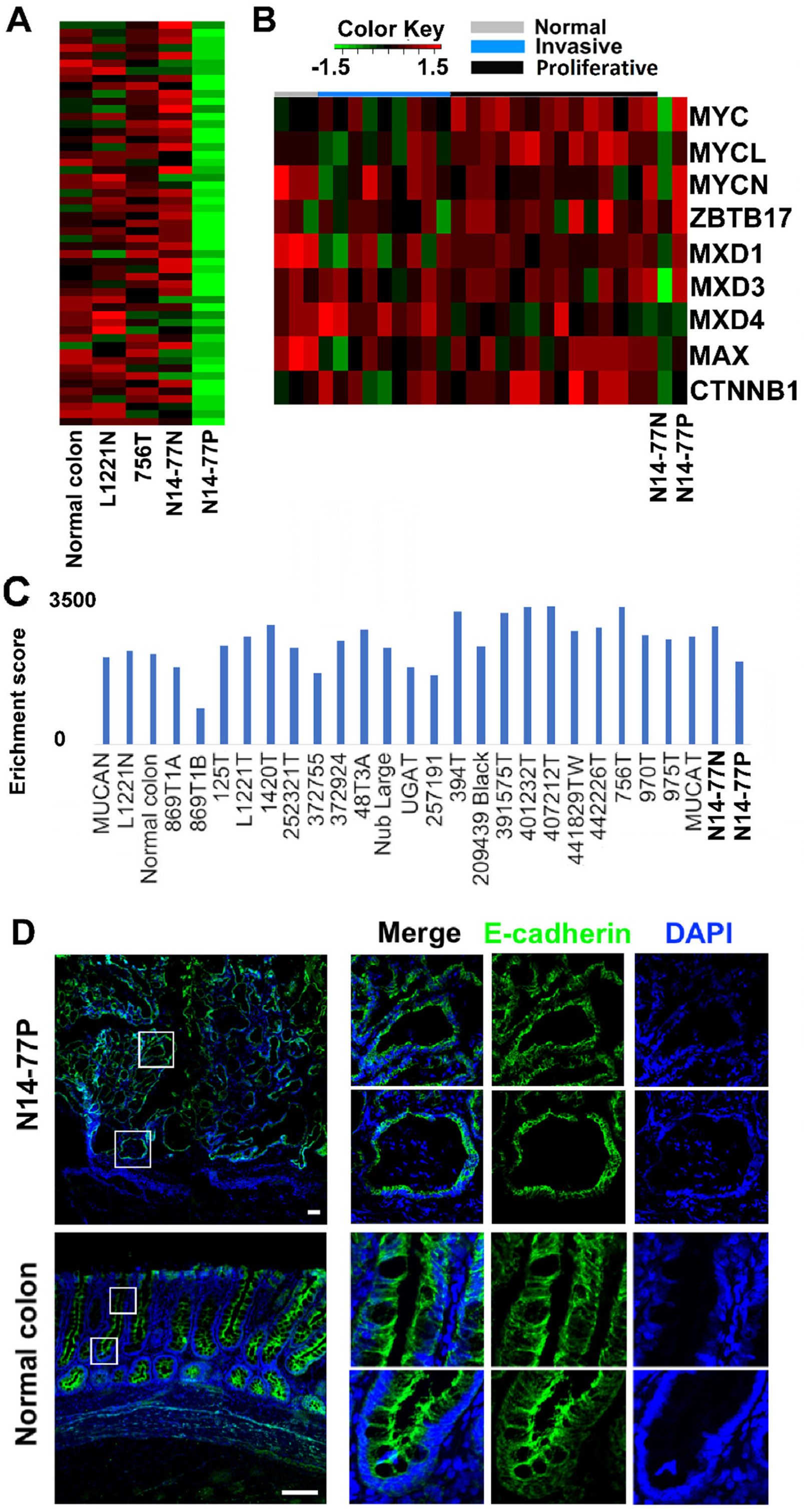

Supplementary Figure 6: Transcriptomic and cell polarity studies of N14-77 polyps, Related to Figures 4, 5 and 7. (A) Heatmap of the $\log_2$ (*FPKM*) values of 53 ubiquitin genes that are lowly expressed in N14-77P (Supplementary Table 4A). **(B)** Heatmap of the $\log_2$ (*FPKM*) values of *MYC,* and some of its co-activators, co-repressors and target genes (Supplementary Table 5A). **(C)** ssGSEA enrichment scores of TGFβ-signaling genes, from both BIOCARTA TGFβ pathway and TGFβ signaling pathway of PMID_23584090, of N14-77 polyp and other canine samples. **(D)** IHC analysis of E-cadherin, an epithelial cell polarity marker, with N14-77 polyp and normal colon tissue samples. Scale bar, 100 μm.

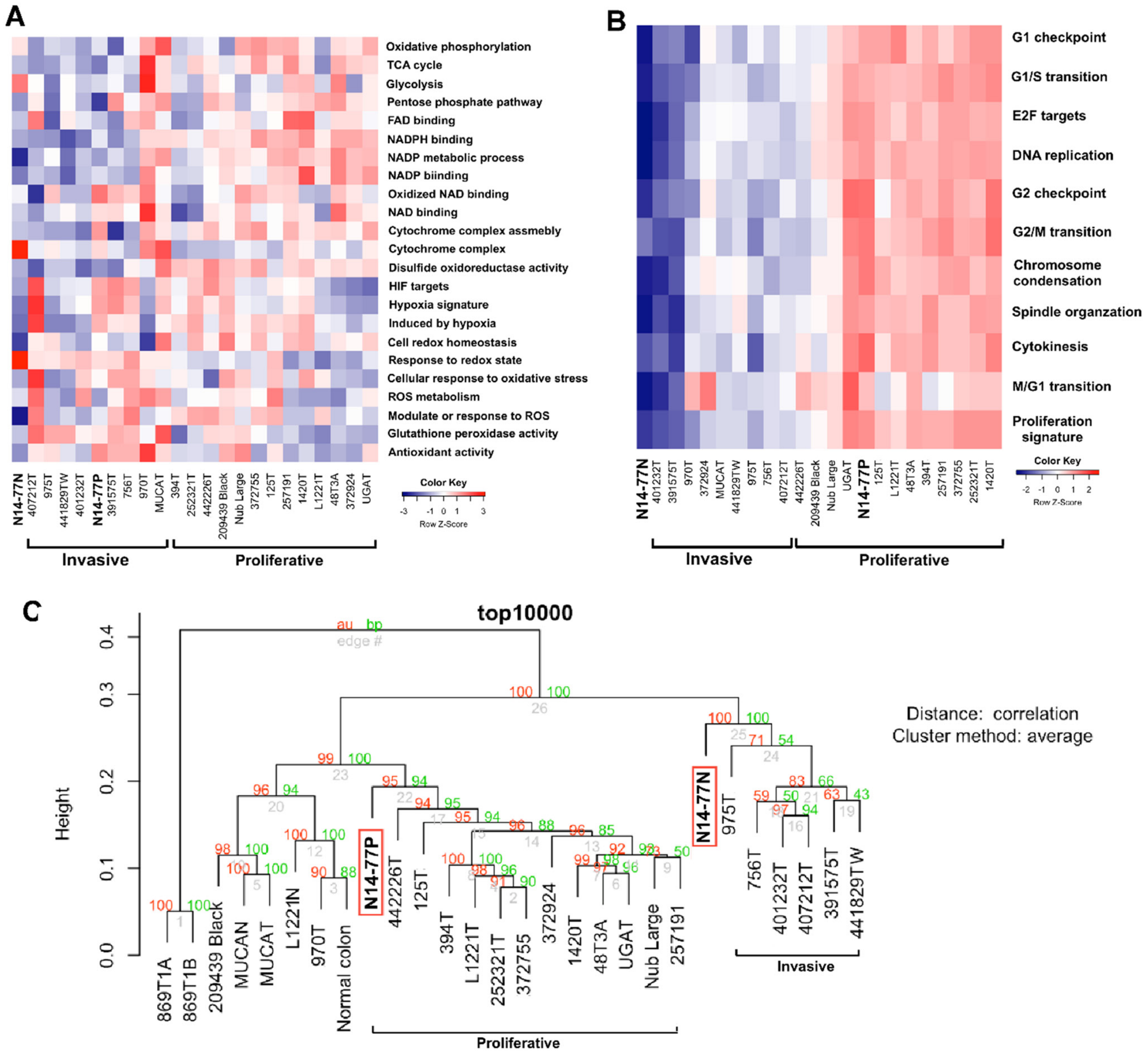

Supplementary Figure 7: N14-77 polyps resemble invasive tumors in redox gene expression while being proliferative, Related to Figure 6. **(A)** Heatmaps of ssGSEA enrichment scores of tissue redox related gene sets (Supplementary Table 6E). **(B)** Heatmaps of ssGSEA enrichment scores of cell cycle and proliferation signature genes (Supplementary Table 6E). **(C)** Hierarchical clustering of canine intestinal samples with top 10,000 most variable genes in expression level.

Supplementary Table 1: WGS and RNA seq sequencing and mapping summary and canine sample information, Related to Figure 1

See Supplementary File 1

Supplementary Table 2: Five canine APC isoforms, Related to Figure 2

See Supplementary File 1

Supplementary Table 3: Germline and somatic mutation findings, Related to Figure 3

See Supplementary File 1

Supplementary Table 4: Highly- and lowly expressed genes, Related to Figure 4

See Supplementary File 1

Supplementary Table 5: Activation of MYC network and crypt proliferative progenitor signature, Related to Figure 5

See Supplementary File 1

Supplementary Table 6: Bacterial phylum, family, species and strain enrichment and gene expression, Related to Figure 6

See Supplementary File 1
